# Machine learning-based prediction of ischemic cardio-cerebrovascular events after endovascular or microsurgical treatment of unruptured intracranial aneurysms and risk stratification by the early post-treatment triglyceride-glucose index

**DOI:** 10.3389/fneur.2026.1829149

**Published:** 2026-05-13

**Authors:** Yingchao He, Shuheng Chen, Deshan Liu, Zheng Zheng, Yongkun Li, Yinzhou Wang

**Affiliations:** 1 Shengli Clinical Medical College of Fujian Medical University, Fuzhou, China; 2 Department of Neurology, Fuzhou University Affiliated Provincial Hospital, Fuzhou, Fujian, China; 3 Fujian Key Laboratory of Medical Analysis, Fujian Academy of Medical Sciences, Fuzhou, China

**Keywords:** CatBoost, ischemic cardio-cerebrovascular events, machine learning, risk stratification, triglyceride-glucose index, unruptured intracranial aneurysm

## Abstract

**Background:**

Ischemic cardio-cerebrovascular events (ICCEs), including acute coronary syndrome and ischemic cerebral infarction, remain clinically important complications after endovascular or microsurgical treatment of unruptured intracranial aneurysms (UIAs). However, early identification of patients at high post-treatment ischemic risk remains challenging, and reliable risk-stratification tools are lacking.

**Objective:**

To develop a machine learning-based framework for predicting ischemic cardio-cerebrovascular events (ICCEs) within 6 months after treatment in patients with unruptured intracranial aneurysms (UIAs) and to evaluate the risk-stratification value of the early post-treatment triglyceride-glucose (TyG) index.

**Methods:**

A total of 1,954 patients with UIAs who underwent microsurgical or endovascular treatment between December 2021 and December 2024 were enrolled from the China Treatment Trial for Unruptured Intracranial Aneurysm (ChTUIA) registry. Nine predictive models, including logistic regression as a baseline comparator, were evaluated after feature selection using least absolute shrinkage and selection operator regression and the Boruta algorithm. The synthetic minority over-sampling technique was used to address class imbalance. Model performance was assessed by discrimination, calibration, and clinical utility metrics, and the optimal model was interpreted using SHapley Additive exPlanations. The association between the post-treatment day-3 TyG index and ICCEs was analyzed using multivariable Cox regression, restricted cubic spline analysis, and subgroup analyses.

**Results:**

During the 6-month follow-up, 240 of 1,954 patients (12.28%) developed ICCEs. Of the included patients, 1,343 underwent endovascular treatment and 611 underwent microsurgical treatment. Among all models, CatBoost achieved the best overall performance, with an accuracy of 0.875 and an area under the receiver operating characteristic curve (AUROC) of 0.945 (95% CI, 0.927–0.963). SHAP analysis identified the post-treatment TyG index as one of the most influential predictors. In multivariable analysis, each 1-unit increase in TyG was associated with a 2.61-fold higher hazard of ICCEs (HR = 2.61, 95% CI: 2.29–2.96, *p* < 0.001). Restricted cubic spline analysis showed a nonlinear positive association with a clear threshold effect at approximately TyG = 7.

**Conclusion:**

The CatBoost model demonstrates strong predictive performance for post-treatment ICCEs in UIA patients. The early post-treatment TyG index is independently and nonlinearly associated with ICCE risk and may serve as a simple, practical metabolic marker for individualized perioperative risk stratification.

## Introduction

Unruptured intracranial aneurysms (UIAs) are being detected with increasing frequency because of the widespread use of high-resolution neuroimaging, and their management has gradually evolved from a narrow focus on rupture prevention to a broader strategy of long-term vascular risk management (1–5). Endovascular therapy has become an important treatment option because of its minimally invasive nature and high technical success rates (3, 5). However, perioperative ischemic cardio-cerebrovascular events (ICCEs) remain an important determinant of post-treatment prognosis, with large cohort studies reporting an incidence of approximately 5–15% for early post-treatment ischemic complications (6, 7). Even after successful aneurysm occlusion, acute cerebral infarction or acute coronary syndrome (ACS) may lead to severe disability or death, underscoring the substantial systemic atherosclerotic burden in patients with UIAs and the role of perioperative stress in aggravating endothelial injury and thrombosis (8, 9).

Current clinical guidelines and consensus-based tools, including the PHASES score and the Unruptured Intracranial Aneurysm Treatment Score (UIATS), mainly focus on rupture risk and provide limited guidance for the early identification of post-treatment ischemic events (10–12). Conventional regression-based models are useful for identifying independent predictors, but they may be insufficient to capture the complex and potentially nonlinear relationships among demographic characteristics, comorbidities, aneurysm morphology, and treatment-related factors that jointly influence ischemic risk after UIA treatment (13). In contrast, machine learning approaches are well suited to high-dimensional and heterogeneous clinical data and have shown promising potential in cerebrovascular risk prediction (14, 15). Metabolic dysregulation is a central mechanism in atherosclerosis, and the triglyceride-glucose (TyG) index, an accessible surrogate marker of insulin resistance, has emerged as a promising prognostic indicator in cardiovascular and cerebrovascular disease (16–18). Insulin resistance may promote endothelial dysfunction and plaque instability through activation of inflammatory pathways, enhancement of oxidative stress, and impairment of nitric oxide bioavailability, and previous studies have linked elevated TyG levels to greater vascular risk and adverse outcomes in cardiovascular and cerebrovascular settings (19, 20). However, the prognostic significance of the TyG index in patients undergoing treatment for UIAs, particularly during the early post-treatment period when metabolic disturbances may be amplified by perioperative stress, remains unclear. Therefore, we aimed to develop a machine learning-based framework for predicting post-treatment ICCEs in patients with UIAs and to evaluate the risk-stratification value of the early post-treatment TyG index, thereby providing evidence for more individualized perioperative management.

## Materials and methods

### Study population and data source

**Figure 1:**
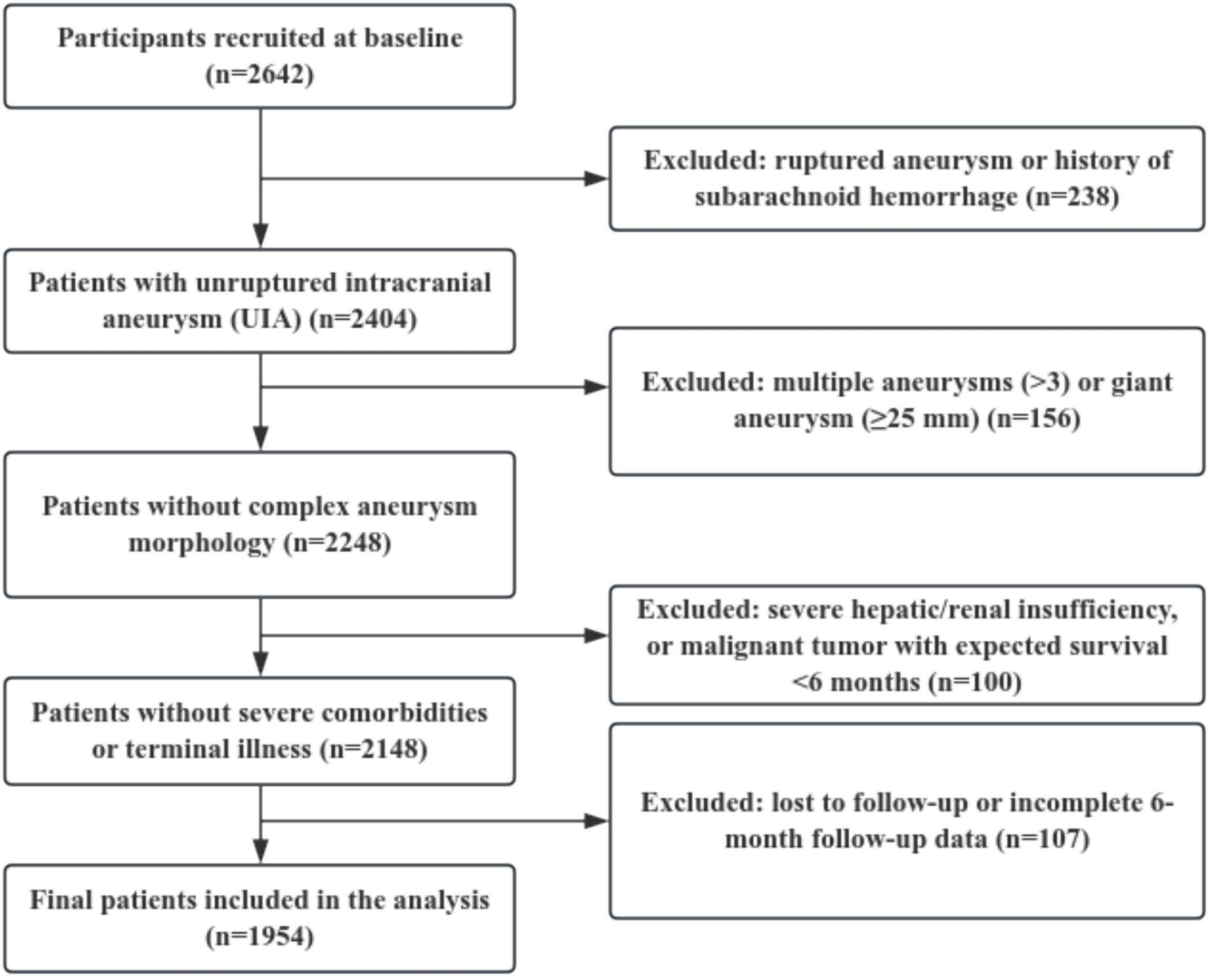
Flowchart of patient enrollment and selection in this study.

This study was based on data from the China Treatment Trial for Unruptured Intracranial Aneurysm (ChTUIA), a national, prospective, observational, multicenter registry study. Clinical data were collected through a standardized electronic data capture (EDC) platform led by Beijing Tiantan Hospital, Capital Medical University (National Clinical Research Center for Neurological Diseases), with participation from regional neuroscience centers including Fujian Provincial Hospital. Patients with unruptured intracranial aneurysms (UIAs) who underwent microsurgical or endovascular treatment between December 2021 and December 2024 were screened for eligibility. The study was approved by the Ethics Committee of Beijing Tiantan Hospital (KY-2022-226) and the Ethics Committee of Fujian Provincial Hospital (K2024-06-041) and was conducted in accordance with the Declaration of Helsinki. Written informed consent was obtained from all participants. All data were de-identified before being uploaded to the centralized database and were managed by an independent data management committee responsible for quality control and data storage. The ChTUIA registry is not publicly available due to institutional governance and patient privacy restrictions. Patient screening flow is illustrated in Figure 1.

### Inclusion and exclusion criteria

Patients were eligible if they met all of the following criteria: (1) age ≥18 years; (2) diagnosis of UIA confirmed by digital subtraction angiography (DSA) or computed tomography angiography (CTA); (3) receipt of microsurgical clipping or endovascular treatment including simple coiling, stent-assisted coiling, or flow-diverter implantation; (4) availability of complete clinical data covering baseline demographic characteristics, medical history, aneurysm morphological features, treatment-related information and post-treatment follow-up records; (5) completion of fasting blood glucose and lipid testing within 3 days after treatment for calculation of the triglyceride-glucose (TyG) index. Patients were excluded if they had any of the following conditions: (1) ruptured aneurysm or a history of subarachnoid hemorrhage; (2) multiple aneurysms (>3) or giant aneurysms (maximum diameter ≥25 mm); (3) severe hepatic or renal insufficiency defined as Child-Pugh class C or an estimated glomerular filtration rate <30 mL/min/1.73 m^2^; (4) a history of malignant tumor or an expected survival <6 months; (5) loss to follow-up or incomplete follow-up data within 6 months after treatment.

### Data collection and variable definitions

#### Baseline variables

Data were independently extracted from the electronic medical records by two trained investigators, and discrepancies were resolved through discussion or adjudication by a third senior reviewer. The collected variables included demographic characteristics (sex and age), lifestyle factors (smoking history and alcohol consumption), medical history (hypertension, diabetes mellitus, dyslipidemia, coronary artery disease and previous cerebral infarction), and medication history (pre-treatment statin use and post-treatment antiplatelet therapy). Smoking was defined as current smoking or smoking cessation within 1 year, and alcohol consumption was defined as drinking at least three times per week. Comorbidities were identified according to medical records and the 10th revision of the International Classification of Diseases (ICD-10) codes. Post-treatment antiplatelet therapy was categorized as no antiplatelet therapy, single antiplatelet therapy or dual antiplatelet therapy.

#### Aneurysm- and treatment-related variables

Aneurysm- and treatment-related variables included aneurysm location, maximum diameter, presence of a daughter sac, intracranial arterial stenosis, treatment modality and specific endovascular techniques. Aneurysm location was classified as anterior or posterior circulation according to hemodynamic characteristics, and clinically significant intracranial arterial stenosis was defined as a stenosis rate ≥50%. Endovascular treatment was further classified as simple coiling, stent-assisted coiling, flow-diverter implantation or other relevant techniques.

#### Laboratory measurements and calculation of the TyG index

The first fasting venous blood sample collected within 3 days after treatment was used for laboratory analysis, and fasting plasma glucose (FPG, mmol/L) and triglyceride (TG, mmol/L) levels were recorded. The TyG index was calculated as TyG = ln [TG (mmol/L) × FPG (mmol/L)/2], according to the prespecified formula used in our analysis. Because TyG values vary according to unit systems and formula conventions, values derived from mmol/L-based and mg/dL-based formulas are not directly interchangeable. Therefore, the apparent threshold identified in this study should be interpreted as unit-specific and cohort-specific.

### Outcome definition and follow-up

The primary outcome was the occurrence of ischemic cardio-cerebrovascular events (ICCEs) within 6 months after treatment, which included two components: (1) acute coronary syndrome (ACS), diagnosed on the basis of clinical symptoms, dynamic electrocardiographic changes, and elevated cardiac biomarkers; and (2) ischemic cerebral infarction, defined as newly detected infarction confirmed by cranial computed tomography or magnetic resonance imaging. ACS events were first clinically confirmed by senior cardiologists according to predefined diagnostic criteria. Thereafter, all study outcomes were independently adjudicated in a blinded manner by two physicians according to a predefined protocol, with disagreements resolved by a senior adjudicator. Follow-up time was calculated from the date of treatment to the first occurrence of an outcome event, 6 months after treatment, or loss to follow-up, whichever came first. Time to event was defined as the number of days from treatment to the first ICCE within 6 months; patients without ICCEs were administratively censored at 180 days.

The composite endpoint of ICCEs, including acute coronary syndrome and ischemic cerebral infarction, was adopted as the primary outcome for the following clinical and methodological reasons. First, both events share overlapping pathophysiological mechanisms in patients with unruptured intracranial aneurysms, including systemic atherosclerosis, endothelial dysfunction, insulin resistance, and perioperative prothrombotic stress (21, 22). Second, both events represent serious acute ischemic complications that strongly influence short-term prognosis and perioperative management strategy. Third, using a composite endpoint increases statistical power and facilitates clinical risk stratification for the overall burden of major acute ischemic events after treatment (23). Nevertheless, we recognize the clinical heterogeneity between the two components and therefore performed comprehensive component-specific analyses to ensure robustness and interpretability.

### Statistical analysis

All statistical analyses were performed using R software (version 4.2.0) and Python (version 3.13). All tests were two-sided, and a *p* value <0.05 was considered statistically significant.

### Baseline characteristic analysis

Patients were divided into the event group and non-event group according to whether ICCEs occurred within 6 months after treatment. The normality of continuous variables was assessed using the Shapiro–Wilk test. Normally distributed continuous variables were presented as mean ± standard deviation and compared using the independent-samples t-test, while non-normally distributed continuous variables were presented as median (interquartile range) and compared using the Mann–Whitney U test. Categorical variables were presented as number (percentage) and compared using the chi-square test or Fisher’s exact test as appropriate. Baseline characteristics are summarized in Table 1.

**Table 1:** Baseline characteristics of patients with and without 6-month ischemic cardio-cerebrovascular events (ICCEs).

Variable	No ICCEs group (*n* = 1,714)	ICCEs group (*n* = 240)	*p* value
Age (years)	66.49 ± 10.83	65.76 ± 10.33	0.31
TyG index at 3 days postoperative	7.26 ± 0.83	8.11 ± 0.91	<0.001
Sex, *n* (%)			0.193
Female	873 (50.93%)	133 (55.42%)	
Male	841 (49.07%)	107 (44.58%)	
Smoking history, *n* (%)			0.013
No	1,411 (82.32%)	213 (88.75%)	
Yes	303 (17.68%)	27 (11.25%)	
Alcohol consumption history, *n* (%)			<0.001
No	1,467 (85.59%)	230 (95.83%)	
Yes	247 (14.41%)	10 (4.17%)	
History of cerebral infarction, *n* (%)			0.224
No	964 (56.24%)	125 (52.08%)	
Yes	750 (43.76%)	115 (47.92%)	
History of diabetes mellitus, *n* (%)			<0.001
No	1,405 (81.97%)	164 (68.33%)	
Yes	309 (18.03%)	76 (31.67%)	
History of hypertension, *n* (%)			0.011
No	1,318 (76.90%)	202 (84.17%)	
Yes	396 (23.10%)	38 (15.83%)	
History of hyperlipidemia, *n* (%)			0.693
No	670 (39.09%)	97 (40.42%)	
Yes	1,044 (60.91%)	143 (59.58%)	
History of coronary heart disease, *n* (%)			0.029
No	1,009 (58.87%)	159 (66.25%)	
Yes	705 (41.13%)	81 (33.75%)	
History of statin use, *n* (%)			<0.001
No	774 (45.16%)	159 (66.25%)	
Yes	940 (54.84%)	81 (33.75%)	
Aneurysm location, *n* (%)			<0.001
Anterior communicating artery	498 (29.05%)	87 (36.25%)	
Internal carotid artery	478 (27.89%)	47 (19.58%)	
Posterior circulation	296 (17.27%)	27 (11.25%)	
Middle cerebral artery	442 (25.79%)	79 (32.92%)	
Aneurysm size, *n* (%)			0.003
10–15 mm	609 (35.53%)	83 (34.58%)	
7–10 mm	691 (40.32%)	122 (50.83%)	
<7 mm	314 (18.32%)	28 (11.67%)	
>15 mm	100 (5.83%)	7 (2.92%)	
Aneurysm daughter sac, *n* (%)			0.001
No	1,628 (94.98%)	239 (99.58%)	
Yes	86 (5.02%)	1 (0.42%)	
Concomitant cerebrovascular stenosis, *n* (%)			<0.001
No	1708 (99.65%)	216 (90.00%)	
Yes	6 (0.35%)	24 (10.00%)	
Surgical approach, *n* (%)			<0.001
Endovascular treatment	1,240 (72.35%)	103 (42.92%)	
Open surgery	474 (27.65%)	137 (57.08%)	
Postoperative antiplatelet therapy, *n* (%)			<0.001
None	676 (39.44%)	149 (62.08%)	
Single antiplatelet	633 (36.93%)	56 (23.33%)	
Dual antiplatelet	405 (23.63%)	35 (14.58%)	

### Machine learning model development

#### Data preprocessing and feature selection

**Figure 2:**
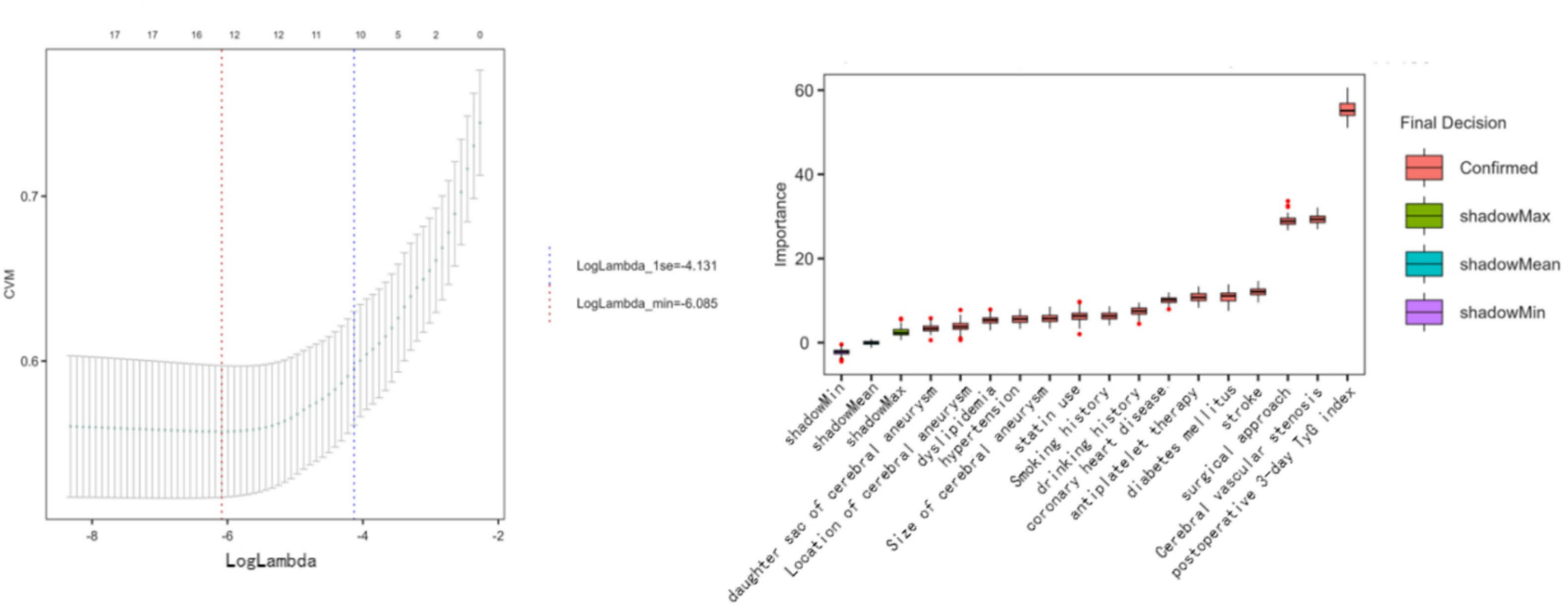
Feature selection using LASSO regression and Boruta algorithm.

Least absolute shrinkage and selection operator (LASSO) regression and the Boruta algorithm were used for feature selection, and the optimal penalty parameter *λ* in LASSO analysis was determined using 10-fold cross-validation. All feature selection procedures were performed exclusively in the training set to avoid data leakage, and the final predictive features were identified by taking the intersection of variables selected by Boruta and LASSO to reduce overfitting and improve model interpretability (24). Results of feature selection are shown in Figure 2.

#### Dataset splitting and class imbalance handling

The enrolled patients were randomly divided into a training set and a test set at a ratio of 8:2 with a random seed of 42 to ensure reproducibility. Given the relatively low incidence of ICCEs, the synthetic minority over-sampling technique (SMOTE) was applied only to the training set to generate minority-class samples using the k-nearest neighbors algorithm and mitigate class imbalance, while the test set retained the original class distribution to provide an unbiased assessment of model performance (25).

#### Machine learning algorithms and hyperparameter settings

A total of 9 predictive models were constructed in this study, including logistic regression as a baseline comparator. All models used default software hyperparameters or basic configurations, with the random seed fixed at 1 to ensure reproducibility. Logistic Regression was performed without regularization, with penalty = ‘none’, solver = ‘lbfgs’, C = 1.0 and maximum iterations of 100. Multi-Layer Perceptron (MLP) was structured with 3 hidden layers containing 100 neurons each, using the Adam optimizer, an initial learning rate of 0.001, L2 regularization strength of 0.0001 and maximum iterations of 200. Support Vector Machine (SVM) was implemented with an RBF kernel and regularization parameter C = 1.0. Decision Tree was set with a maximum depth of 3, split criterion of ‘gini’, minimum samples per split of 2 and minimum samples per leaf of 1. LightGBM (LGBM) adopted GBDT as the base learner with a learning rate of 0.1. Random Forest (RF) consisted of 100 decision trees, with maximum depth of 3, split criterion of ‘gini’, minimum samples per split of 2 and minimum samples per leaf of 1. Naive Bayes (NB) was performed as Multinomial Naive Bayes with Laplace smoothing parameter alpha = 1.0 and no fitted class priors. CatBoost was trained with logloss objective, 100 iterations, maximum tree depth of 10 and learning rate of 0.1. KNN (k-nearest neighbors) was implemented with n_neighbors = 5, weights = ‘uniform’, algorithm = ‘auto’, leaf_size = 30, *p* = 2, and metric = ‘minkowski’. No complex hyperparameter grid search was performed to ensure fair comparison and stability across models.

#### Model performance evaluation

**Figure 3:**
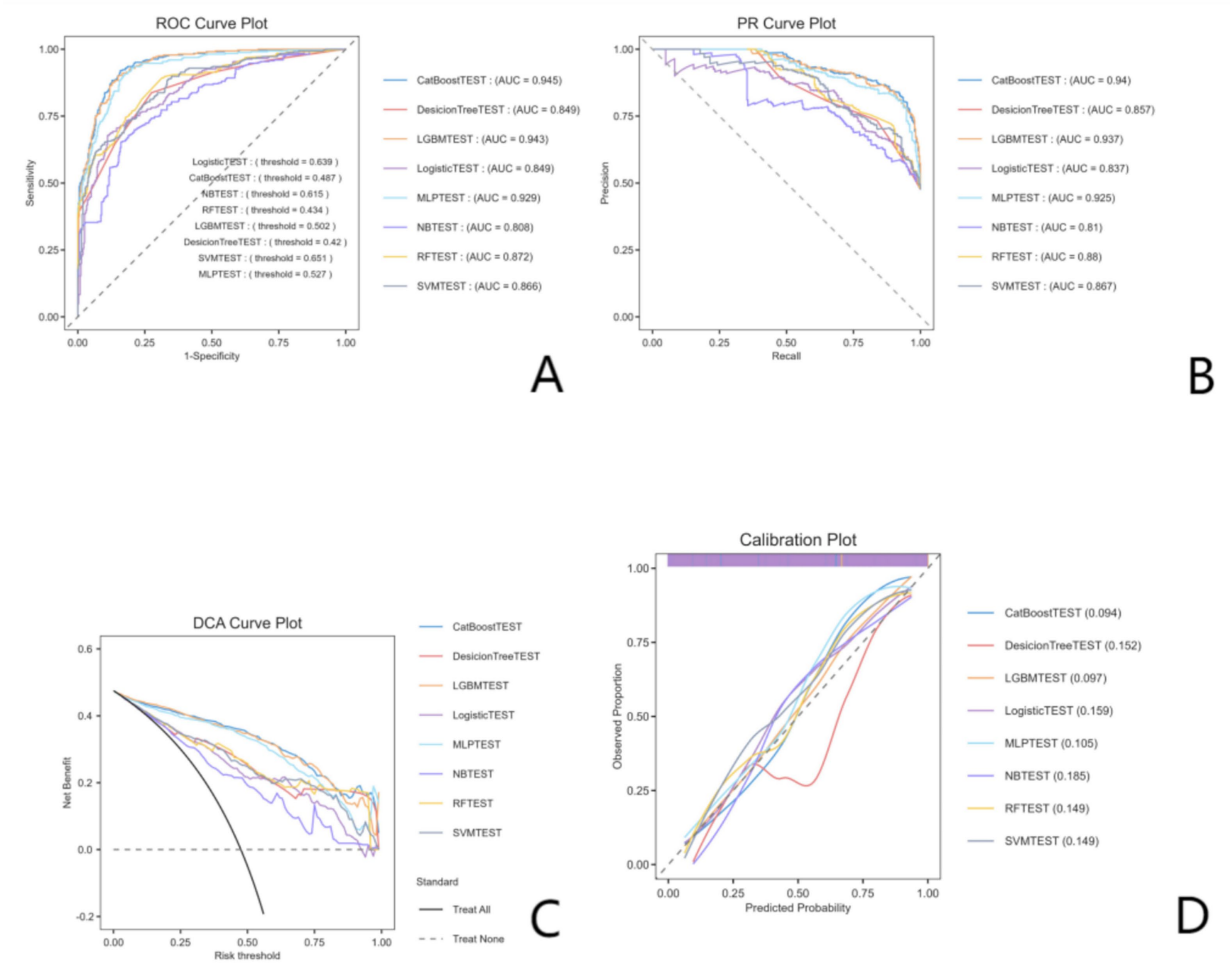
Comprehensive performance evaluation of predictive models. **(A)** Receiver operating characteristic (ROC) curves and corresponding area under the curve (AUC) values for all models, evaluating their discriminative ability to distinguish between positive and negative outcomes. **(B)** Precision-recall (PR) curves and AUC values, providing additional assessment of model performance, especially in the context of imbalanced outcome distributions. **(C)** Decision curve analysis (DCA) curves, assessing the clinical net benefit of each model across a range of risk thresholds, with reference lines for the “treat all” and “treat none” strategies. **(D)** Calibration curves, evaluating the agreement between predicted probabilities and observed actual outcomes, with Brier scores (in parentheses) indicating the overall prediction accuracy (lower scores represent better calibration). AUC, area under the curve; DCA, decision curve analysis; PR, precision-recall; ROC, receiver operating characteristic.

Model performance was comprehensively evaluated using discrimination metrics (AUROC, AUPRC), classification metrics (accuracy, sensitivity, specificity, precision, F1 score) and calibration metrics (Brier score, calibration intercept and slope), and clinical utility was assessed using decision curve analysis (DCA). Ten-fold cross-validation was performed with mean ± standard deviation reported for AUROC and accuracy, as detailed in Supplementary Table S1 and illustrated in Supplementary Figure S1. A total of 1,000 iterations of bootstrap validation were applied for optimism correction and internal stability assessment, with metrics shown in Supplementary Table S2. Performance metrics of all models on the test set are listed in Table 2 and Supplementary Table S3. Comprehensive performance evaluation, including ROC curves, precision-recall curves, calibration plots, and DCA, is presented in Figure 3. No *post hoc* probability calibration (e.g., Platt scaling or isotonic regression) was performed.

**Table 2:** Performance metrics of predictive models on the test set.

Model name	Accuracy	Recall	F1-Score	MCC	AUROC (95% CI)	Precision	Specificity	FNR	FPR
MLPTEST	0.859	0.914	0.86	0.723	0.929 (0.908–0.949)	0.812	0.808	0.086	0.192
SVMTEST	0.771	0.785	0.765	0.543	0.866 (0.838–0.894)	0.746	0.758	0.215	0.242
DecisionTreeTEST	0.778	0.837	0.782	0.564	0.849 (0.820–0.879)	0.734	0.725	0.163	0.275
LGBMTEST	0.873	0.902	0.871	0.748	0.943 (0.924–0.961)	0.842	0.847	0.098	0.153
RFTEST	0.783	0.739	0.764	0.564	0.872 (0.845–0.900)	0.790	0.822	0.261	0.178
NBTEST	0.717	0.773	0.722	0.441	0.808 (0.775–0.841)	0.677	0.667	0.227	0.333
CatBoostTEST	0.875	0.911	0.874	0.752	0.945 (0.926–0.963)	0.839	0.842	0.089	0.158
LogisticTEST	0.742	0.791	0.745	0.489	0.849 (0.819–0.878)	0.703	0.697	0.209	0.303
KNNTEST	0.894	0.935	0.899	0.79	0.928 (0.907–0.948)	0.866	0.852	0.065	0.148

AUROC values are presented with approximate 95% confidence intervals estimated using the Hanley–McNeil method. AUROC, area under the receiver operating characteristic curve; MCC, Matthews correlation coefficient; FNR, false negative rate; FPR, false positive rate.

#### SHAP interpretability analysis

**Figure 4:**
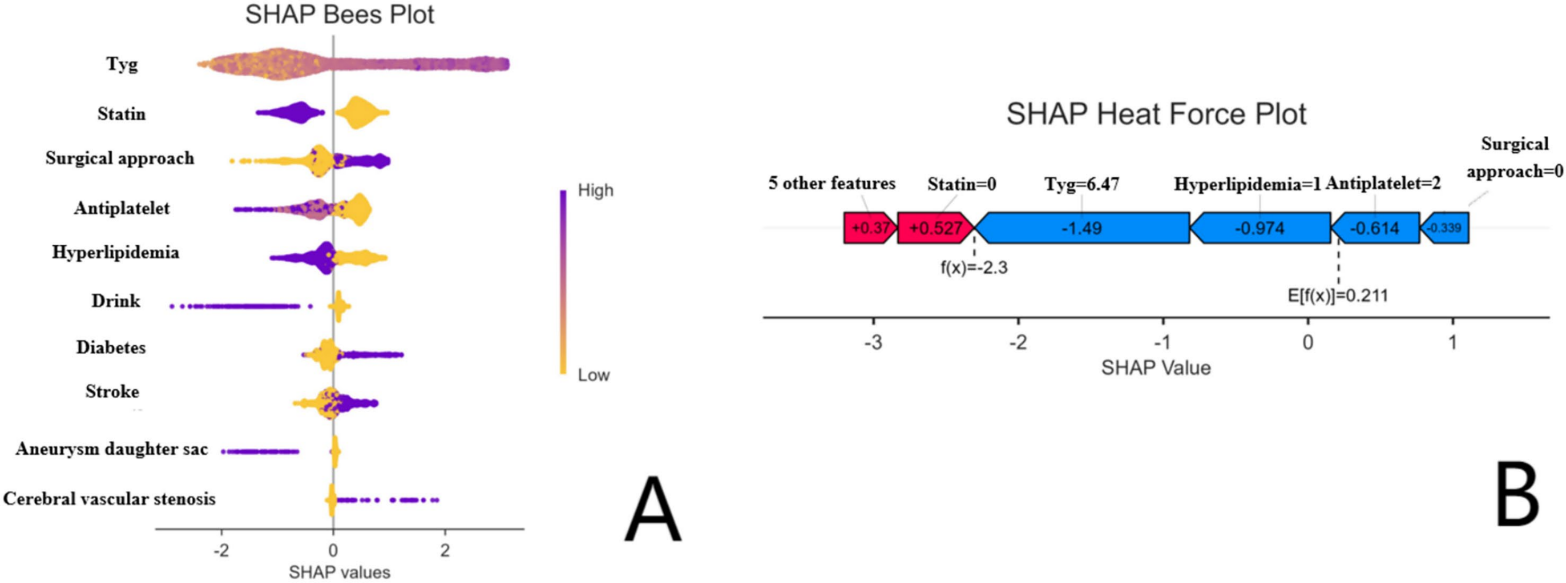
SHAP analysis of the CatBoost model. **(A)** SHAP Bees Plot showing feature importance and directionality of each variable. **(B)** SHAP Heat Force Plot illustrating the contribution of individual features to the prediction for a specific case.

SHapley Additive exPlanations (SHAP) was used to interpret the best-performing CatBoost model based on Shapley values from cooperative game theory, which quantifies the contribution of each feature to an individual prediction (26). Global SHAP values were calculated to rank feature importance, and SHAP beeswarm and force plots were generated to visualize the direction and magnitude of feature effects on predicted risk, as shown in Figure 4.

### Analysis of the risk-stratification value of the TyG index

#### Trend analysis

Patients were divided into four groups (Q1–Q4) according to quartiles of the TyG index, and the Cochran-Armitage trend test was used to assess the linear trend between TyG quartiles and the incidence of ICCEs with corresponding P for trend calculated.

#### Restricted cubic spline analysis

**Figure 5:**
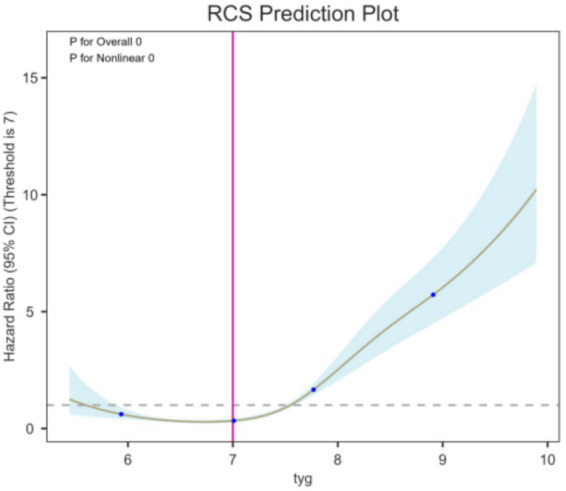
Restricted cubic spline analysis of the TyG index and ICCE risk.

Restricted cubic spline (RCS) analysis was performed to explore the nonlinear association between the TyG index (as a continuous variable) and the risk of ICCEs within 6 months after treatment (27). Four knots were placed at the 5th, 35th, 65th and 95th percentiles with the median TyG value used as the reference. The model was adjusted for potential confounders including age, sex, diabetes mellitus, hypertension, aneurysm size and treatment modality. A dose–response curve was generated, and the statistical significance of nonlinearity was evaluated using the likelihood ratio test (Figure 5). Separate RCS analyses were conducted for ACS alone (Supplementary Figure S2), ischemic cerebral infarction alone (Supplementary Figure S3), endovascular treatment subgroup (Supplementary Figure S4), microsurgical treatment subgroup (Supplementary Figure S5), Beijing Tiantan Hospital subgroup (Supplementary Figure S6) and Fujian Provincial Hospital subgroup (Supplementary Figure S7).

#### Time-to-event analysis

Considering the 6-month follow-up with clear event time records, Cox proportional hazards regression was used as the primary inferential method instead of logistic regression. The TyG index was analyzed as both a continuous variable and a categorical variable by quartiles in the Cox model, with stepwise adjustment for demographics, comorbidities, aneurysm characteristics, treatment modality and antiplatelet therapy. Results are presented in Table 3; Supplementary Tables S4, S5.

**Table 3:** Multivariable cox regression analysis of the early postoperative TyG index and 6-month postoperative ischemic events in UIA patients.

TyG index at postoperative 3 days	Model 1 HR (95%CI)	Model 2 HR (95%CI)	Model 3 HR (95%CI)
Per 1-unit increase (continuous)	2.587 (2.270, 2.948)	2.674 (2.336, 3.061)	2.605 (2.292, 2.960)
Quartiles of TyG index			
Q1 (reference)	1	1	1
Q2	1.705 (0.953, 3.050)	1.730 (0.967, 3.095)	1.754 (0.981, 3.135)
Q3	1.691 (0.952, 3.003)	1.896 (1.061, 3.388)	1.92 (1.085, 3.401)
Q4	9.390 (5.751 15.331)	9.943 (6.02, 16.309)	10.292 (6.318, 16.764)
*P* for trend	<0.001	<0.001	<0.001

Model 1: adjusted for age, sex, smoking status, and alcohol consumption; Model 2: further adjusted for hypertension, diabetes mellitus, and dyslipidemia based on Model 1; Model 3: further adjusted for aneurysm location (anterior/posterior circulation), maximum diameter (mm), presence of daughter sac, intracranial artery stenosis (≥50%), surgical approach (endovascular/open craniotomy), and postoperative antiplatelet therapy (none/single/dual antiplatelet therapy) based on Model 2.

#### Interaction and stratified analysis

**Figure 6:**
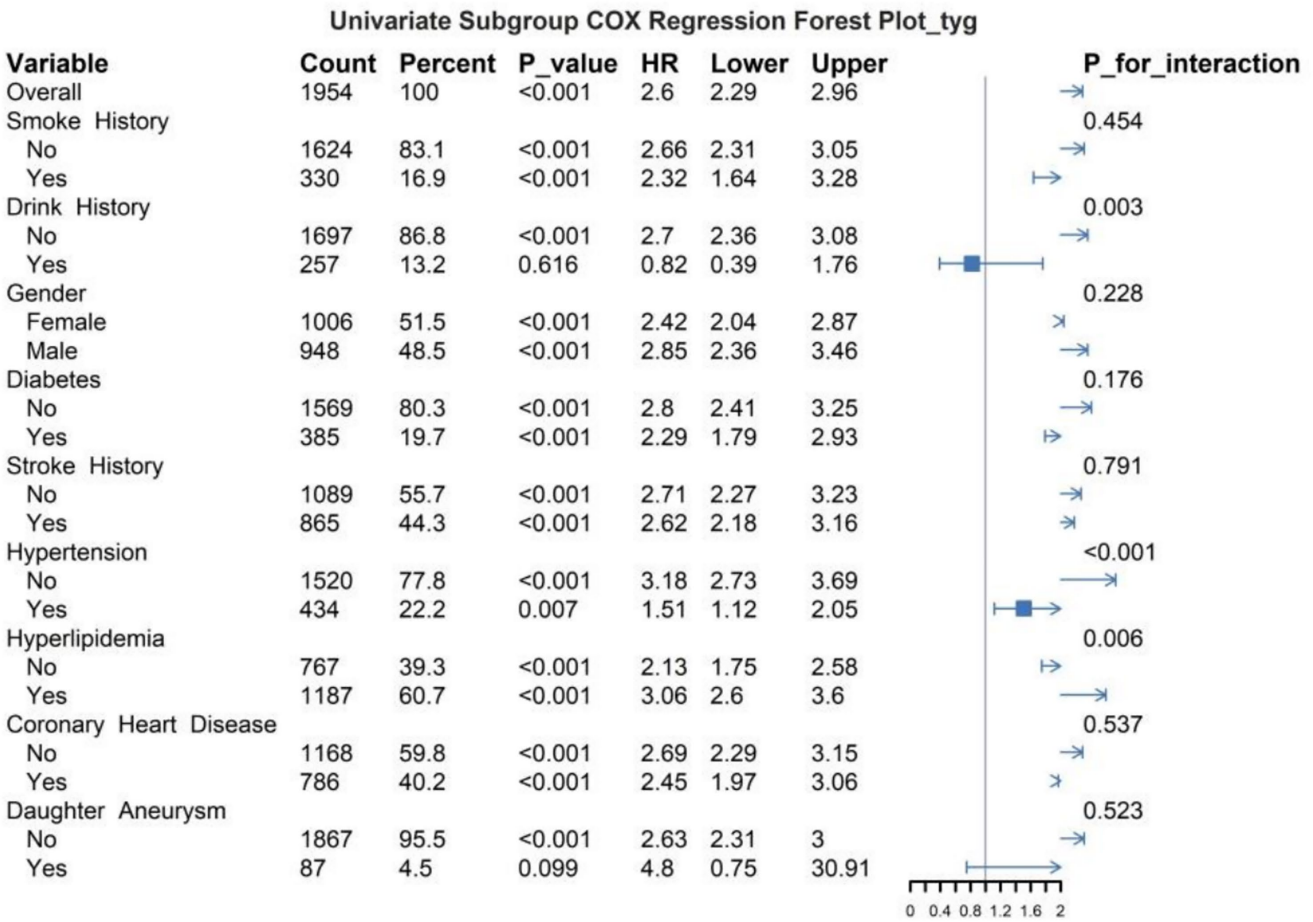
Forest plot of subgroup analyses for the association between the TyG index and ICCEs.

An interaction term was constructed by assigning endovascular treatment as 0 and microsurgical treatment as 1, no antiplatelet therapy as 0, single antiplatelet therapy as 1 and dual antiplatelet therapy as 2, then calculating the product of treatment modality and antiplatelet therapy. The interaction term, treatment modality and antiplatelet therapy were simultaneously incorporated into the Cox model to test the interaction effect (Supplementary Table S6). Stratified analyses were performed by treatment modality (endovascular vs. microsurgical) and by center (Beijing Tiantan Hospital vs. Fujian Provincial Hospital). Subgroup analyses were conducted using Cox regression with interaction *p* values reported, and results are displayed in a forest plot (Figure 6). The distribution of postoperative antiplatelet therapy by treatment modality is shown in Table 4.

**Table 4:** Distribution of post-treatment antiplatelet therapy by treatment modality.

Variable	Level	Endovascular treatment (*n* = 1,343)	Open surgery (*n* = 611)	*p*-value
Postoperative antiplatelet therapy	None	392 (29.19)	433 (70.87)	<0.001
	Single antiplatelet	527 (39.24)	162 (26.51)	
	Dual antiplatelet	424 (31.57)	16 (2.62)	

#### Component-specific outcome analysis

Separate CatBoost models and RCS analyses were established for ischemic cerebral infarction alone and ACS alone. Component-specific model performance is summarized in Supplementary Table S7, and corresponding ROC, PR, calibration, and DCA curves are shown in Supplementary Figure S8. Center-specific CatBoost model performance is listed in Supplementary Table S8, with corresponding curves in Supplementary Figure S9.

#### Quality control and reporting standard

Several measures were implemented to ensure data quality and analytical rigor, including independent double data extraction with cross-checking and inconsistency verification, blinded outcome adjudication, strict separation of training and test sets to avoid data leakage, independent third-party review of analytic code for reproducibility, and reported in line with the TRIPOD+AI guidance for clinical prediction model studies using machine-learning methods.

## Results

### Baseline characteristics and event distribution

A total of 1,954 patients with unruptured intracranial aneurysms (UIAs) who underwent microsurgical or endovascular treatment were included in the analysis. Patient enrollment and selection flow are presented in Figure 1. During the 6-month follow-up period, 240 patients (12.28%) developed ischemic cardio-cerebrovascular events (ICCEs), including 131 cases of acute coronary syndrome (ACS) and 109 cases of ischemic cerebral infarction, while 1,714 patients remained event-free. Baseline characteristics between patients with and without ICCEs were compared in Table 1. No significant differences were observed in age or sex between the two groups. Compared with the non-event group, patients who developed ICCEs had a significantly higher early post-treatment TyG index and a higher prevalence of diabetes mellitus. In contrast, smoking history, regular alcohol consumption, hypertension, and a history of coronary artery disease were less common in the event group. For aneurysm- and treatment-related factors, patients in the event group were more likely to have anterior communicating artery aneurysms, concomitant intracranial arterial stenosis, and microsurgical treatment, and were less likely to receive post-treatment antiplatelet therapy or statins.

### Feature selection for predictive modeling

Least absolute shrinkage and selection operator (LASSO) regression and the Boruta algorithm were jointly applied to identify robust predictors of ICCEs. The intersection of the two methods yielded 10 variables for model development, as shown in Figure 2, including alcohol consumption, previous stroke, diabetes mellitus, dyslipidemia, statin use, the post-treatment day-3 TyG index, aneurysm daughter sac, intracranial arterial stenosis, treatment modality, and post-treatment antiplatelet therapy.

### Performance of predictive models for 6-month ICCEs

#### Model comparison and main performance

Nine predictive models, including logistic regression as a baseline comparator, were trained and tested for predicting 6-month ICCEs using the selected features. Among all models, the CatBoost model achieved the best overall performance on the test set, with an area under the receiver operating characteristic curve (AUROC) of 0.945 (95% CI: 0.926–0.963), an area under the precision-recall curve (AUPRC) of 0.937, and a low Brier score of 0.097, as shown in Table 2 and Supplementary Table S3. It also outperformed the other models across key metrics, including accuracy (0.875), recall (0.911), F1-score (0.874), Matthews correlation coefficient (MCC, 0.752), and specificity (0.842), with a low false negative rate (FNR, 0.089) and false positive rate (FPR, 0.158). The LightGBM model demonstrated comparable performance to CatBoost, with an AUROC of 0.943 (95% CI: 0.924–0.961), accuracy of 0.873, and MCC of 0.748, while the KNN model also achieved strong discriminative ability (AUROC: 0.928, 95% CI: 0.907–0.948) and the highest accuracy (0.894) and recall (0.935) among all models. Although the CatBoost model showed excellent discrimination and a low Brier score, the calibration slope and intercept indicated that calibration was not optimal. The logistic regression baseline model showed an AUROC of 0.849 (95% CI: 0.819–0.878) and an AUPRC of 0.840, further supporting the incremental predictive value of the machine learning approach. Comprehensive evaluation of the top-performing models, including receiver operating characteristic (ROC) curves, precision-recall (PR) curves, calibration plots, and decision curve analysis (DCA), is shown in Figure 3.

#### Model stability: cross-validation and bootstrap validation

Ten-fold cross-validation demonstrated stable discriminative performance of the CatBoost model, with detailed metrics shown in Supplementary Table S1 and corresponding ROC curves illustrated in Supplementary Figure S1. Bootstrap validation (1,000 iterations) further confirmed the internal stability and low optimism bias of the CatBoost model, with key indicators listed in Supplementary Table S2.

#### SHAP interpretability

SHapley Additive exPlanations (SHAP) analysis revealed the top five influential predictors in the CatBoost model, as illustrated in Figure 4, including the post-treatment TyG index, post-treatment antiplatelet therapy, intracranial arterial stenosis, treatment modality, and diabetes mellitus. Higher TyG values, no antiplatelet therapy, presence of arterial stenosis, and microsurgical treatment were associated with increased predicted risk of ICCEs.

### Association between the post-treatment TyG index and ICCEs

#### Overall association and dose–response relationship

Multivariable Cox proportional hazards regression showed that the post-treatment day-3 TyG index was independently associated with ICCEs, as detailed in Table 3. In the fully adjusted model, each 1-unit increase in the TyG index was associated with a 2.61-fold higher hazard of ICCEs (hazard ratio [HR], 2.61; 95% CI, 2.29–2.96; *p* < 0.001). When analyzed by quartiles, a significant dose–response trend was observed (*P* for trend < 0.001), with patients in the highest TyG quartile showing a 10.29-fold increased hazard compared with those in the lowest quartile. Restricted cubic spline (RCS) analysis demonstrated a significant non-linear positive association between the TyG index and ICCE risk (*P* for non-linearity < 0.001), and the hazard ratio increased sharply when the TyG index exceeded approximately 7, suggesting a clinically relevant threshold effect, as displayed in Figure 5.

#### Component-specific analysis: ACS vs. ischemic cerebral infarction

To account for the composite nature of ICCEs, separate analyses were performed for ACS and ischemic cerebral infarction. The TyG index was significantly associated with both outcomes, with corresponding dose–response curves shown in Supplementary Figures S2, S3. The CatBoost model maintained high discriminative performance for each component, with performance metrics summarized in Supplementary Table S7 and comprehensive evaluation curves presented in Supplementary Figures S8.

#### Stratified analyses by treatment modality

The distribution of post-treatment antiplatelet therapy by treatment modality is summarized in Table 4. Post-treatment antiplatelet therapy varied significantly between patients undergoing endovascular versus microsurgical treatment. Patients in the microsurgical group were far less likely to receive dual antiplatelet therapy and more likely to receive no antiplatelet therapy. The association between the TyG index and ICCEs stratified by treatment modality is shown in Supplementary Table S4, with corresponding RCS curves displayed in Supplementary Figures S4, S5. The interaction analysis between treatment modality and antiplatelet therapy is presented in Supplementary Table S6. The association between the TyG index and ICCEs was stronger in the microsurgical group than in the endovascular group. The interaction term between treatment modality and antiplatelet therapy was not statistically significant in the Cox model. However, microsurgical treatment itself was independently associated with a higher hazard of ICCEs, while single antiplatelet therapy was associated with a lower hazard compared with no antiplatelet therapy.

#### Sensitivity analysis by participating center

To assess the generalizability of findings across centers, analyses were stratified by participating hospital (Beijing Tiantan Hospital vs. Fujian Provincial Hospital). The prognostic value of the TyG index remained consistent in both cohorts, as detailed in Supplementary Table S5, with corresponding RCS curves shown in Supplementary Figures S6, S7. Center-specific CatBoost model performance is listed in Supplementary Table S8, with comprehensive evaluation curves presented in Supplementary Figure S9.

#### Subgroup analysis of the TyG-ICCE association

The association between the TyG index and ICCEs was generally consistent across most pre-specified subgroups, including sex, smoking status, previous stroke, diabetes mellitus, coronary artery disease, and presence of an aneurysm daughter sac. However, significant effect modification was observed for alcohol consumption, hypertension, and dyslipidemia. The results of subgroup analyses with interaction tests are visualized in Figure 6.

(1) The association was not statistically significant among patients with regular alcohol consumption (HR = 0.82; 95% CI: 0.39–1.76; *p* = 0.616). (2) The magnitude of association was weaker in patients with hypertension (HR = 1.51; 95% CI: 1.12–2.05; *p* = 0.007) than in those without hypertension (HR = 3.18; 95% CI: 2.73–3.69; *p* < 0.001; *P* for interaction < 0.001). (3) The association was stronger in patients with dyslipidemia (HR = 3.06; 95% CI: 2.60–3.60; *p* < 0.001) than in those without (HR = 2.13; 95% CI: 1.75–2.58; *p* < 0.001; *P* for interaction = 0.006).

## Discussion

In this multicenter observational cohort of patients treated for UIAs, we developed and assessed a machine learning–based framework for predicting 6-month post-treatment ICCEs and explored the value of the early post-treatment TyG index for risk stratification. Three key findings emerged. First, ICCEs occurred in 12.28% of patients during follow-up, underscoring the clinical importance of post-treatment ischemic vascular complications in this population. Second, among the nine machine learning models evaluated, CatBoost demonstrated the best overall predictive performance, along with satisfactory robustness and interpretability. Third, the post-treatment day-3 TyG index was independently associated with ICCE risk in both continuous and categorical analyses, and restricted cubic spline analysis revealed a significant nonlinear positive relationship. Collectively, these findings indicate that risk assessment following UIA treatment should move beyond aneurysm occlusion alone and integrate comprehensive vascular and metabolic vulnerability. Although ACS and ischemic cerebral infarction were analyzed as a clinically relevant composite ischemic endpoint for fixed-window prediction, their temporal profiles were not identical. In our cohort, the median time to event was 67 days for ACS and 75 days for ischemic cerebral infarction, supporting the value of component-wise reporting while preserving the clinical relevance of the 6-month composite outcome.

A key implication of this study is that technically successful treatment of UIAs does not completely resolve residual cerebrovascular and cardiovascular risk. In current clinical practice, decision-making for UIAs remains largely guided by rupture-focused strategies centered on aneurysm size, location, and morphology (28). Our results demonstrate that post-treatment ischemic events are shaped by a wider array of factors, including systemic vascular comorbidities, intracranial arterial stenosis, metabolic status, treatment modality, and perioperative management. This broader view is especially relevant for modern UIA cohorts, where aging, diabetes mellitus, dyslipidemia, and subclinical atherosclerosis are highly prevalent. From a neurological perspective, these patients remain at risk of thromboembolic cerebral infarction or ACS even after technically successful aneurysm treatment. Thus, post-treatment evaluation would benefit from shifting from a lesion-centered framework to a holistic assessment of the patient’s overall vascular vulnerability.

In this context, our findings support the clinical value of machine learning for post-treatment risk stratification in neurovascular care. Conventional regression models remain useful for detecting independent predictors but often fail to capture nonlinear effects and complex interactions among demographic, metabolic, anatomical, and treatment-related variables. By comparison, machine learning methods are well equipped to integrate multidimensional clinical data and uncover latent risk patterns that conventional models cannot easily detect. This is particularly relevant for UIA patients, in whom post-treatment ICCEs are rarely driven by a single factor but arise from interactions between aneurysm features, procedural factors, antiplatelet management, and systemic vascular vulnerability. In our study, CatBoost achieved the strongest discriminative performance among all models, and cross-validation confirmed acceptable stability. Furthermore, SHAP analysis improved model transparency by identifying the post-treatment TyG index, post-treatment antiplatelet therapy, intracranial arterial stenosis, treatment modality, and diabetes mellitus as the leading predictive factors. These results enhance the clinical interpretability of the model and support its potential utility in postoperative neurovascular management.

Another critical finding is the strong prognostic value of the post-treatment day-3 TyG index. The TyG index is widely accepted as a practical surrogate marker for insulin resistance and metabolic dysfunction (29, 30). Previous studies in cardiovascular and cerebrovascular populations have linked elevated TyG levels to atherosclerotic burden, endothelial dysfunction, platelet activation, ischemic stroke, and adverse cardiovascular outcomes (31–35). More recently, the TyG index has also been associated with clinical outcomes in patients with aneurysmal subarachnoid hemorrhage (36). However, those studies differ considerably from the present work in clinical setting, timing of TyG measurement, and outcome definition. Unlike earlier reports focused on the natural history of aneurysms or post-hemorrhagic prognosis, our study specifically included patients treated for UIAs, used 6-month post-treatment ICCEs as the primary endpoint, and centered on the post-treatment day-3 TyG index rather than only baseline metabolic status. In this respect, our study expands the growing body of TyG-related research into post-treatment vascular risk stratification for UIA patients.

Our results indicate that TyG can serve as a valuable adjunctive marker for identifying patients at elevated short-term ischemic risk after UIA treatment. In fully adjusted models, the association between TyG and ICCEs remained significant when TyG was analyzed as both a continuous variable and a categorical variable by quartiles. Moreover, patients in the highest TyG quartile had a markedly higher hazard of ICCEs than those in the lowest quartile, suggesting that post-treatment metabolic status provides prognostic information beyond conventional clinical variables. This association is biologically plausible. Insulin resistance is closely associated with vascular inflammation, oxidative stress, endothelial dysfunction, impaired fibrinolysis, and platelet hyperreactivity (29, 30, 35). In the perioperative period, these abnormalities can be further exacerbated by procedural stress, endothelial injury from endovascular or microsurgical treatment, hemodynamic fluctuations, and altered responsiveness to antiplatelet therapy. Patients with elevated post-treatment TyG levels may therefore represent a subgroup with both chronic metabolic vulnerability and enhanced acute thromboinflammatory activation, which partially explains the persistent association between TyG and ICCEs after multivariable adjustment (32–34).

Restricted cubic spline analysis further reinforces the clinical relevance of TyG. Instead of a simple linear relationship, the spline model showed a nonlinear positive association, with the hazard of ICCEs increasing more steeply at higher TyG levels. This pattern suggests that TyG may act as a threshold-dependent marker: mild elevations carry limited incremental prognostic value, while higher levels identify a subgroup with significantly increased post-treatment vulnerability. Clinically, this implies that TyG is especially useful for detecting a metabolically high-risk subgroup rather than being interpreted only as a continuous biochemical indicator. This finding also supports the use of quartile- or cutoff-based stratification in future post-treatment risk models, although clinically validated thresholds require further investigation.

Subgroup analyses offer additional, albeit nuanced, evidence for the prognostic role of TyG. Overall, the association between higher TyG levels and ICCEs remained directionally consistent across most predefined subgroups. However, significant interactions were detected for alcohol consumption, hypertension, and dyslipidemia, indicating that the strength of the association may vary based on metabolic and vascular comorbidity status. These findings suggest that TyG does not provide a uniform risk signal across all subgroups; instead, its prognostic impact is modified by the patient’s underlying clinical context. Because these subgroup analyses were based on Cox regression within a predefined 6-month follow-up period, the observed associations should be interpreted as subgroup-specific hazard estimates rather than purely descriptive between-group differences.

Several baseline observations warrant cautious interpretation. The event group had a higher prevalence of diabetes mellitus and higher post-treatment TyG index than the non-event group, both consistent with increased vascular risk. In contrast, several traditional vascular risk factors—including smoking history, regular alcohol consumption, hypertension, and coronary artery disease—were less common in the event group. These patterns should be interpreted carefully. Given the observational study design, such findings likely reflect treatment selection bias, residual confounding, variation in documentation, or differences in baseline medical management rather than true protective effects. These variables should not be overinterpreted in isolation and must be considered within the broader multivariable and model-based framework of this study.

Our findings have practical implications for post-treatment management. If externally validated, the machine learning model and TyG-based risk stratification tool could help identify patients who need closer monitoring, more intensive optimization of vascular risk factors, or more rigorous neurological and cardiovascular follow-up after UIA treatment. In particular, patients with elevated post-treatment TyG levels represent a subgroup in whom metabolic status deserves greater attention during the perioperative and early follow-up periods. Importantly, these findings should not be taken as support for selecting any specific treatment strategy based solely on TyG. Instead, they reinforce the value of integrating metabolic markers into a comprehensive post-treatment vascular risk assessment framework (28).

## Limitations

First, despite a registry-based design, data were collected mainly from two centers, which may limit generalizability and introduce selection bias. Second, the model was only validated internally; external validation is needed to confirm clinical applicability. Third, TyG was measured only at postoperative day 3 and may be affected by perioperative stress, so it should be interpreted as a prognostic marker rather than a causal factor. Fourth, detailed information regarding antiplatelet therapy timing and adherence was incomplete, representing a potential confounder. Fifth, as an observational study, residual confounding and treatment-selection bias could not be fully eliminated. Although ACS and cerebral infarction represent distinct clinical entities, the consistent directionality and robust predictive performance observed in component-specific analyses support the clinical validity of the composite ICCE endpoint for capturing overall post-treatment vascular vulnerability.

## Conclusion

In conclusion, the early post-treatment TyG index was independently and nonlinearly associated with 6-month ischemic cardio-cerebrovascular event risk in patients undergoing treatment for unruptured intracranial aneurysms. Among the nine machine learning models tested, the CatBoost model achieved the best predictive performance, calibration, and clinical interpretability for risk stratification. These findings highlight the importance of integrating metabolic status into postoperative vascular risk evaluation and support a shift from a lesion-centered to a comprehensive, whole-patient management strategy for UIA patients. Future external validation in large-scale multicenter cohorts is warranted to verify model generalizability, establish standardized TyG cutoff values, and explore whether TyG-guided perioperative strategies can reduce post-treatment ischemic events and improve clinical outcomes.

## Data Availability

The original contributions presented in the study are included in the article/Supplementary material, further inquiries can be directed to the corresponding author.
