# Supplementary material for: Machine learning-based prediction of ischemic cardio-cerebrovascular events after endovascular or microsurgical treatment of unruptured intracranial aneurysms and risk stratification by the early post-treatment triglyceride-glucose index
Source: Front Neurol. 2026 May 13;17:1829149. doi: 10.3389/fneur.2026.1829149 (PMC13212117; doi:10.3389/fneur.2026.1829149)
Supplement: Supplementary file 1 [file Table_1.DOCX]

**Table S1. Ten-fold cross-validation performance metrics of the CatBoost model**

| **Fold** | **Accuracy** | **Prevalence** | **Recall** | **F1-Score** | **MCC** | **AUROC** | **Precision** | **Specificity** | **FNR** | **FPR** |
| --- | --- | --- | --- | --- | --- | --- | --- | --- | --- | --- |
| Fold_1 | 0.879 | 0.108 | 0.412 | 0.424 | 0.357 | 0.853 | 0.438 | 0.936 | 0.588 | 0.064 |
| Fold_2 | 0.892 | 0.096 | 0.467 | 0.452 | 0.392 | 0.885 | 0.438 | 0.937 | 0.533 | 0.063 |
| Fold_3 | 0.854 | 0.178 | 0.536 | 0.566 | 0.479 | 0.802 | 0.600 | 0.922 | 0.464 | 0.078 |
| Fold_4 | 0.878 | 0.096 | 0.400 | 0.387 | 0.32 | 0.793 | 0.375 | 0.929 | 0.600 | 0.071 |
| Fold_5 | 0.885 | 0.083 | 0.615 | 0.471 | 0.425 | 0.827 | 0.381 | 0.909 | 0.385 | 0.091 |
| Fold_6 | 0.897 | 0.199 | 0.677 | 0.724 | 0.664 | 0.909 | 0.778 | 0.952 | 0.323 | 0.048 |
| Fold_7 | 0.865 | 0.122 | 0.684 | 0.553 | 0.490 | 0.857 | 0.464 | 0.891 | 0.316 | 0.109 |
| Fold_8 | 0.846 | 0.147 | 0.609 | 0.538 | 0.452 | 0.896 | 0.483 | 0.887 | 0.391 | 0.113 |
| Fold_9 | 0.923 | 0.09 | 0.571 | 0.571 | 0.529 | 0.864 | 0.571 | 0.958 | 0.429 | 0.042 |
| Fold_10 | 0.878 | 0.109 | 0.529 | 0.486 | 0.420 | 0.820 | 0.450 | 0.921 | 0.471 | 0.079 |
| MEAN | 0.880 | 0.123 | 0.550 | 0.517 | 0.453 | 0.851 | 0.498 | 0.924 | 0.45 | 0.076 |
| STD | 0.022 | 0.039 | 0.101 | 0.096 | 0.097 | 0.039 | 0.122 | 0.023 | 0.101 | 0.023 |

Ten-fold cross-validation was performed to evaluate the internal stability and discrimination of the CatBoost model. Abbreviations: AUROC, area under the receiver operating characteristic curve; MCC, Matthews correlation coefficient; FNR, false negative rate; FPR, false positive rate; Mean, average value; STD, standard deviation.

**Table S2. Bootstrap validation metrics of the CatBoost model (1,000 iterations)**

| Metric | Bootstrap Mean | SD | Min | Max | 95% CI | Optimism |
| --- | --- | --- | --- | --- | --- | --- |
| Accuracy | 0.833 | 0.008 | 0.802 | 0.855 | (0.815, 0.847) | ＜0.001 |
| Recall | 0.839 | 0.024 | 0.747 | 0.906 | (0.790, 0.882) | ＜0.001 |
| F1-Score | 0.834 | 0.009 | 0.794 | 0.86 | (0.813, 0.850) | ＜0.001 |
| MCC | 0.666 | 0.016 | 0.603 | 0.712 | (0.634, 0.695) | ＜0.001 |
| AUROC | 0.911 | 0.006 | 0.89 | 0.929 | (0.899, 0.921) | ＜0.001 |
| Precision | 0.829 | 0.015 | 0.769 | 0.873 | (0.801, 0.858) | ＜0.001 |
| Specificity | 0.826 | 0.02 | 0.747 | 0.885 | (0.790, 0.864) | ＜0.001 |
| FNR | 0.161 | 0.024 | 0.094 | 0.253 | (0.118, 0.210) | ＜0.001 |
| FPR | 0.174 | 0.02 | 0.115 | 0.253 | (0.136, 0.210) | ＜0.001 |

Bootstrap validation with 1,000 resampling iterations was performed to assess internal model stability, calibration optimism, and potential overfitting. No obvious optimism bias or overfitting was observed.Abbreviations: SD, standard deviation; CI, confidence interval; AUROC, area under the receiver operating characteristic curve; MCC, Matthews correlation coefficient; FNR, false negative rate; FPR, false positive rate.

**Table S3. Calibration performance metrics of machine learning models for predicting 6-month ischemic cardio-cerebrovascular events in patients with unruptured intracranial aneurysms**

| **Model** | **AUPRC** | **Brier Score** | **Calibration Intercept** | **Calibration Slope** |
| --- | --- | --- | --- | --- |
| MLP | 0.8975 | 0.1225 | 0.5218 | 0.0694 |
| SVM | 0.8668 | 0.1499 | 0.4548 | 0.1759 |
| DecisionTree | 0.7299 | 0.1880 | 0.4587 | 0.0171 |
| LGBM | 0.9366 | 0.0957 | 0.4465 | 0.1005 |
| RF | 0.9260 | 0.103 | 0.4415 | 0.0304 |
| NB | 0.8290 | 0.4316 | 0.3309 | 0.0233 |
| CatBoost | 0.9367 | 0.0974 | 0.4553 | 0.1115 |
| Logistic | 0.8400 | 0.1599 | 0.4715 | 0.1666 |
| KNN | 0.8838 | 0.1017 | 0.4768 | 0.0247 |

AUPRC, area under the precision-recall curve; Brier Score, measures the accuracy of probabilistic predictions (lower values indicate better calibration); Calibration Intercept, ideal value is 0 (values >0 indicate underestimation of risk, values <0 indicate overestimation); Calibration Slope, ideal value is 1 (values <1 indicate overfitting or too extreme predictions, values >1 indicate underfitting or too conservative predictions); MLP, multilayer perceptron; SVM, support vector machine; LGBM, light gradient boosting machine; CatBoost, categorical boosting; KNN, k-nearest neighbors. The CatBoost and LGBM models demonstrated the best calibration performance with the lowest Brier scores (0.097 and 0.096, respectively) and highest AUPRC values (both 0.937). Calibration intercepts closer to 0 and slopes closer to 1 indicate better model calibration.

**Table S4. Cox regression analysis of the TyG index by treatment modality**

| TyG index | Treatment Modality | Model 1 HR (95%CI) | Model 2 HR (95%CI) | Model 3 HR (95%CI) |
| --- | --- | --- | --- | --- |
| Per 1‑unit increase | Endovascular | 1.678 (1.400–2.012) | 1.710 (1.419–2.061) | 1.699 (1.419–2.034) |
|  | Microsurgical | 10.948 (8.336–14.379) | 10.425 (7.745–14.032) | 10.841 (8.280–14.194) |
| Quartiles of TyG index |  |  |  |  |
| Q1 (reference) | Endovascular | 1 | 1 | 1 |
|  | Microsurgical | 1 | 1 | 1 |
| Q2 | Endovascular | 0.752 (0.352–1.607) | 0.726 (0.339–1.554) | 0.794 (0.372–1.696) |
|  | Microsurgical | 1.006 (0.352–2.870) | 1.105 (0.387–3.159) | 1.008 (0.354–2.875) |
| Q3 | Endovascular | 1.573 (0.824–3.004) | 1.609 (0.842–3.075) | 1.636 (0.858–3.119) |
|  | Microsurgical | 2.682 (1.120–6.424) | 2.898 (1.207–6.959) | 2.719 (1.136–6.510) |
| Q4 | Endovascular | 3.570 (2.005–6.358) | 3.435 (1.927–6.123) | 3.695 (2.080–6.563) |
|  | Microsurgical | 24.375 (11.303–52.565) | 22.912 (10.617–49.445) | 24.325 (11.298–52.373) |
| *P* for trend | Endovascular | <0.001 | <0.001 | <0.001 |
|  | Microsurgical | <0.001 | <0.001 | <0.001 |

Model 1: Adjusted for age, sex, smoking status, and alcohol consumption.Model 2: Further adjusted for hypertension, diabetes mellitus, and dyslipidemia.Model 3: Further adjusted for aneurysm location, maximum diameter, daughter sac, intracranial arterial stenosis, and postoperative antiplatelet therapy.HR, hazard ratio; CI, confidence interval; TyG, triglyceride‑glucose index.

**Table S5. Cox regression analysis of the TyG index by participating center**

| **TyG index** | **Center** | **Model 1 HR (95%CI)** | **Model 2 HR (95%CI)** | **Model 3 HR (95%CI)** |
| --- | --- | --- | --- | --- |
| Per 1‑unit increase | Fujian Provincial Hospital | 2.806 (2.206–3.568) | 2.791 (2.167–3.594) | 2.892 (2.273–3.681) |
|  | Beijing Tiantan Hospital | 2.504 (2.139–2.931) | 2.645 (2.246–3.115) | 2.504 (2.152–2.914) |
| Quartiles of TyG index |  |  |  |  |
| Q1 (reference) | Fujian Provincial Hospital | 1 | 1 | 1 |
|  | Beijing Tiantan Hospital | 1 | 1 | 1 |
| Q2 | Fujian Provincial Hospital | 2.736 (0.724–10.341) | 2.846 (0.752–10.773) | 2.693 (0.714–10.151) |
|  | Beijing Tiantan Hospital | 1.602 (0.839–3.058) | 1.612 (0.844–3.079) | 1.636 (0.858–3.118) |
| Q3 | Fujian Provincial Hospital | 4.474 (1.291–15.498) | 5.076 (1.443–17.855) | 5.247 (1.519–18.124) |
|  | Beijing Tiantan Hospital | 1.026 (0.510–2.063) | 1.152 (0.570–2.329) | 1.134 (0.567–2.271) |
| Q4 | Fujian Provincial Hospital | 18.076 (5.619–58.156) | 18.575 (5.700–60.524) | 19.082 (5.942–61.278) |
|  | Beijing Tiantan Hospital | 7.890 (4.579–13.596) | 8.574 (4.950–14.852) | 8.610 (5.019–14.771) |
| *P* for trend | Fujian Provincial Hospital | <0.001 | <0.001 | <0.001 |
|  | Beijing Tiantan Hospital | <0.001 | <0.001 | <0.001 |

Model 1: Adjusted for age, sex, smoking status, and alcohol consumption.Model 2: Further adjusted for hypertension, diabetes mellitus, and dyslipidemia.Model 3: Further adjusted for aneurysm location, maximum diameter, daughter sac, intracranial arterial stenosis, treatment modality, and postoperative antiplatelet therapy.HR, hazard ratio; CI, confidence interval; TyG, triglyceride‑glucose index; ICCEs, ischemic cardio‑cerebrovascular events.

**Table S6. Cox regression analysis of treatment modality, antiplatelet therapy, and their interaction**

| **Characteristics** | **Category** | **n (%)** | **HR (95%CI)** | ***P-*value** |
| --- | --- | --- | --- | --- |
| Interaction term  (Treatment × Antiplatelet) | 0 | 1776 (90.9) | Reference | — |
|  | 1 | 162 (8.3) | 1.579 (0.839–2.973) | 0.157 |
|  | 2 | 16 (0.8) | 0.689 (0.158–3.000) | 0.62 |
| Postoperative antiplatelet  therapy | None | 825 (42.2) | Reference | — |
|  | Single antiplatelet | 689 (35.3) | 0.446 (0.276–0.722) | 0.001 |
|  | Dual antiplatelet | 440 (22.5) | 0.687 (0.436–1.081) | 0.104 |
| Treatment modality | Endovascular | 1343 (68.7) | Reference | — |
|  | Microsurgical | 611 (31.3) | 2.359 (1.655–3.362) | < 0.001 |

The interaction term was defined as follows: Treatment (Endovascular = 0, Microsurgical = 1) × Antiplatelet therapy (None = 0, Single = 1, Dual = 2). The model was adjusted for age, sex, smoking status, alcohol consumption, hypertension, diabetes mellitus, dyslipidemia, aneurysm location, aneurysm size, daughter sac, and intracranial arterial stenosis.HR, hazard ratio; CI, confidence interval.

**Table S7. Component-specific CatBoost model performance**

| Outcome Component | Ischemic Cerebral Infarction | Acute Coronary Syndrome (ACS) |
| --- | --- | --- |
| Accuracy | 0.8916 | 0.8836 |
| Prevalence | 0.4824 | 0.4836 |
| Recall | 0.8989 | 0.9490 |
| F1-Score | 0.8889 | 0.8874 |
| MCC | 0.7833 | 0.7749 |
| AUROC | 0.9537 | 0.9353 |
| Precision | 0.8791 | 0.8333 |
| Specificity | 0.8848 | 0.8223 |
| FNR | 0.1011 | 0.0510 |
| FPR | 0.1152 | 0.1777 |

Performance metrics of the CatBoost model were evaluated separately for each component of the composite ischemic cardio‑cerebrovascular event (ICCE) outcome.Abbreviations: MCC, Matthews correlation coefficient; AUROC, area under the receiver operating characteristic curve; FNR, false negative rate; FPR, false positive rate.

**Table S8. Center-specific CatBoost model performance**

| Center | Beijing Tiantan Hospital | Fujian Provincial Hospital |
| --- | --- | --- |
| Accuracy | 0.8960 | 0.8829 |
| Prevalence | 0.4969 | 0.5073 |
| Recall | 0.8954 | 0.9423 |
| F1-Score | 0.8954 | 0.8909 |
| MCC | 0.7921 | 0.7707 |
| AUROC | 0.9567 | 0.9687 |
| Precision | 0.8954 | 0.8448 |
| Specificity | 0.8967 | 0.8218 |
| FNR | 0.1046 | 0.0577 |
| FPR | 0.1033 | 0.1782 |

Performance metrics of the CatBoost model for predicting 6‑month ischemic cardio‑cerebrovascular events (ICCEs) were evaluated separately in each center.Abbreviations: MCC, Matthews correlation coefficient; AUROC, area under the receiver operating characteristic curve; FNR, false negative rate; FPR, false positive rate.

**Table S9. Temporal distribution of overall and component-specific ischemic cardio-cerebrovascular events within 6 months after treatment**

| Outcome component | Overall ICCEs | Acute coronary syndrome (ACS) | Ischemic cerebral infarction |
| --- | --- | --- | --- |
| No. of events | 240 | 131 | 109 |
| Median time to event, days (IQR) | 71.0 (36.8–103.0) | 67.0 (41.5–103.0) | 75.0 (34.0–103.0) |
| 0–30 days, n (%) | 46 (19.2) | 23 (17.6) | 23 (21.1) |
| 31–90 days, n (%) | 105 (43.8) | 56 (42.7) | 49 (45.0) |
| 91–180 days, n (%) | 89 (37.1) | 52 (39.7) | 37 (33.9) |

Time to event was defined as the number of days from treatment to the first ischemic cardio-cerebrovascular event (ICCE) within 6 months after treatment. Percentages were calculated within each outcome component.

**
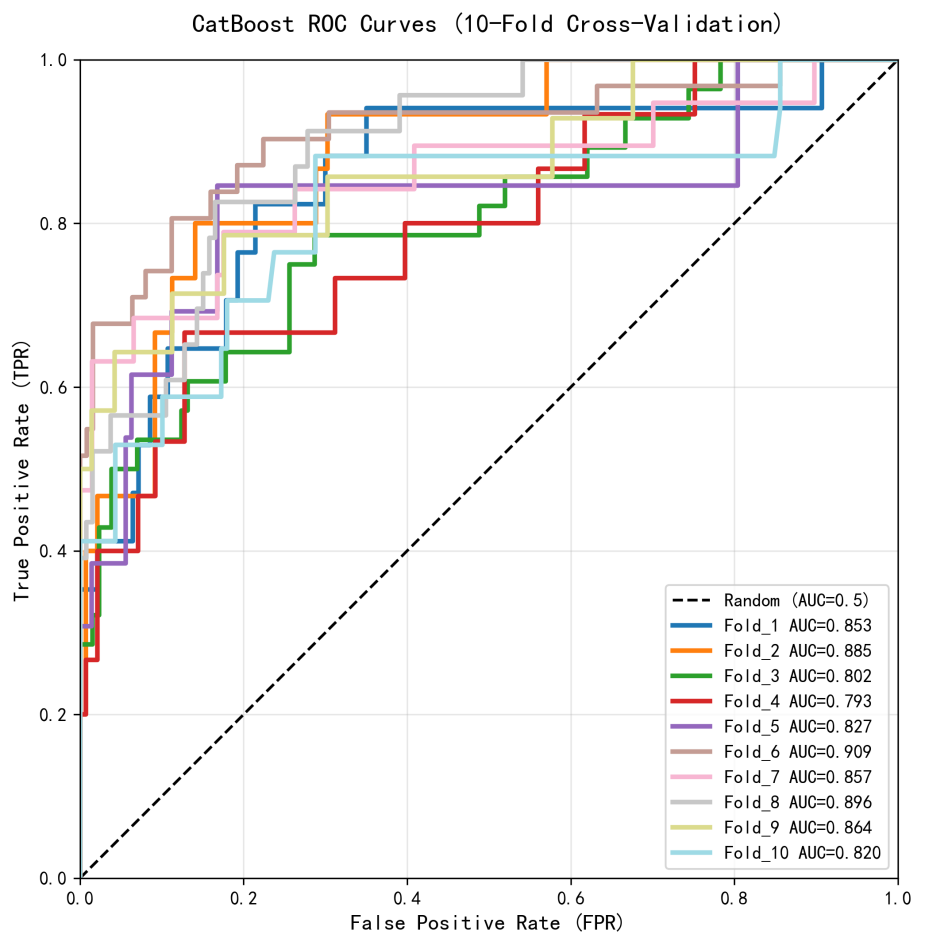
**

**Figure S1. Ten-fold cross-validation ROC curves of the CatBoost model**

**
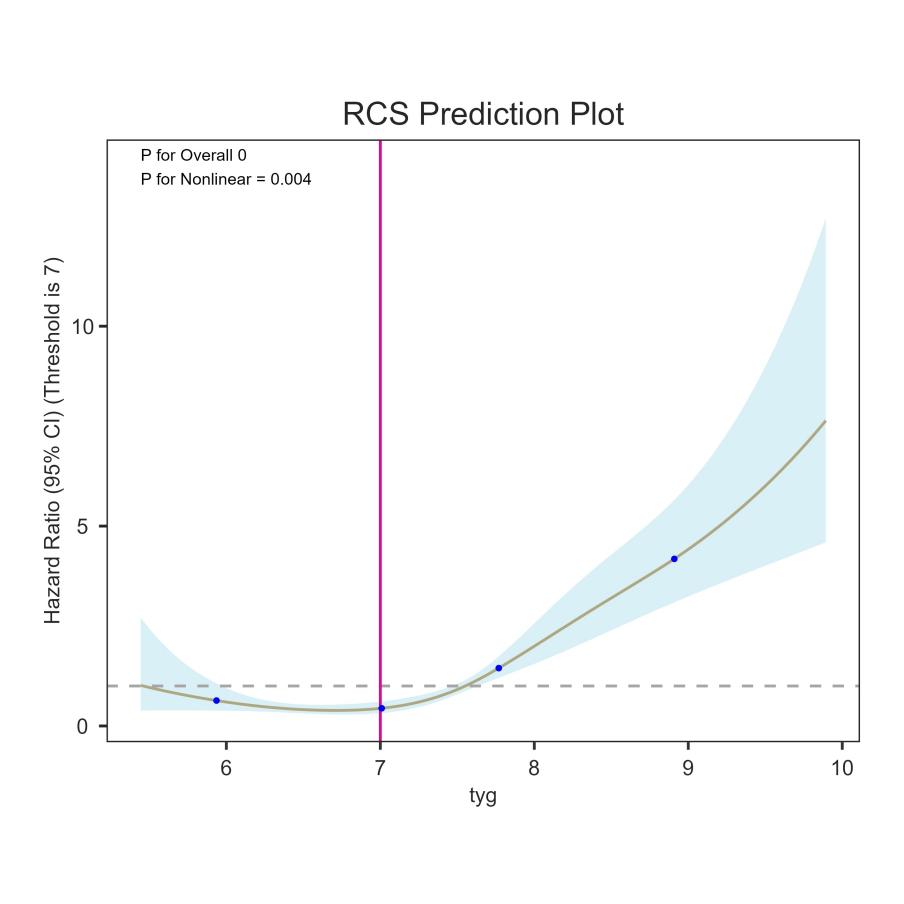
**

**Figure S2. RCS analysis of the TyG index for ACS**

**
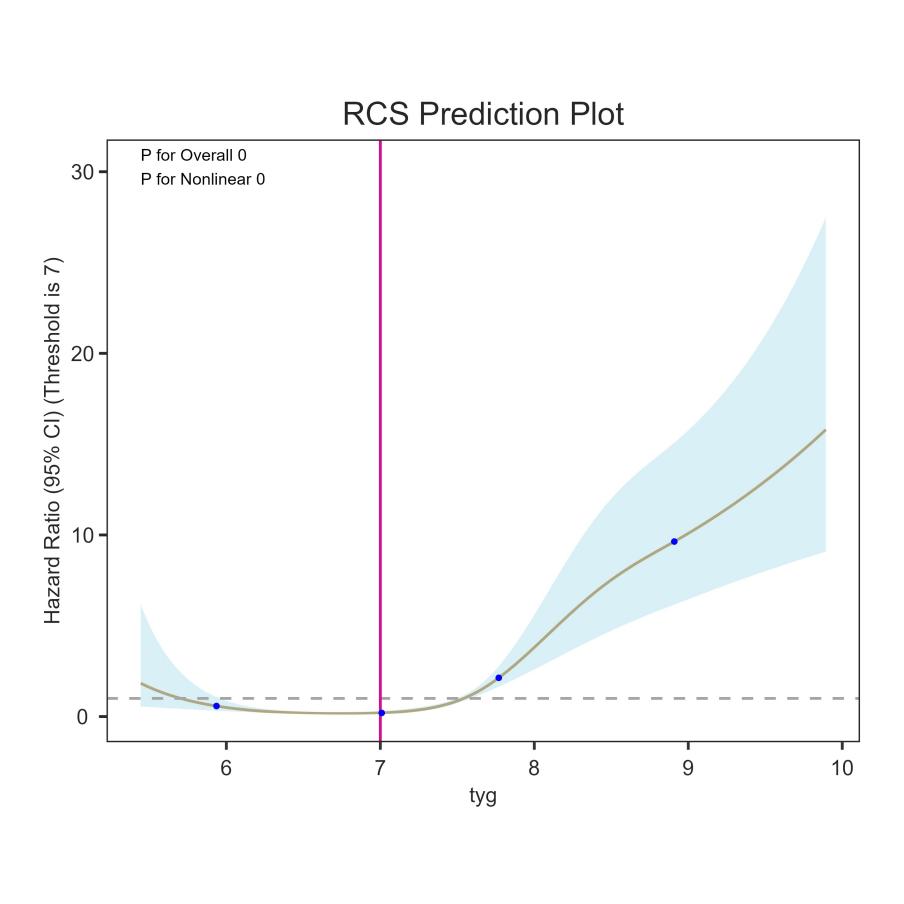
**

**Figure S3. RCS analysis of the TyG index for ischemic cerebral infarction**

**
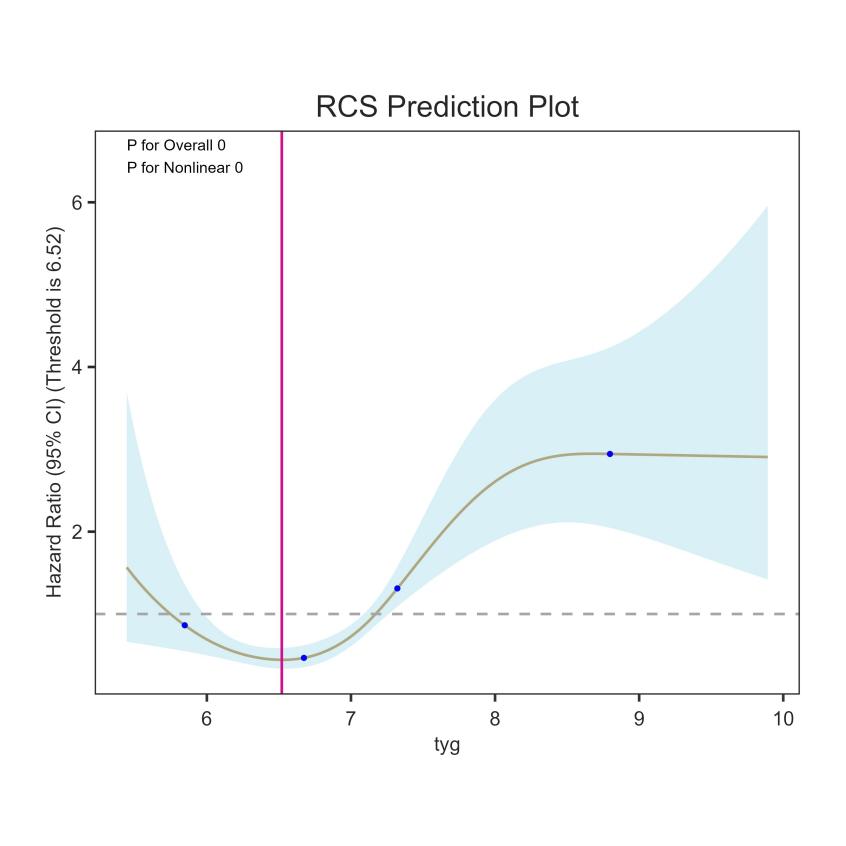
**

**Figure S4. RCS analysis of the TyG index and ICCE risk in the endovascular treatment group**

**
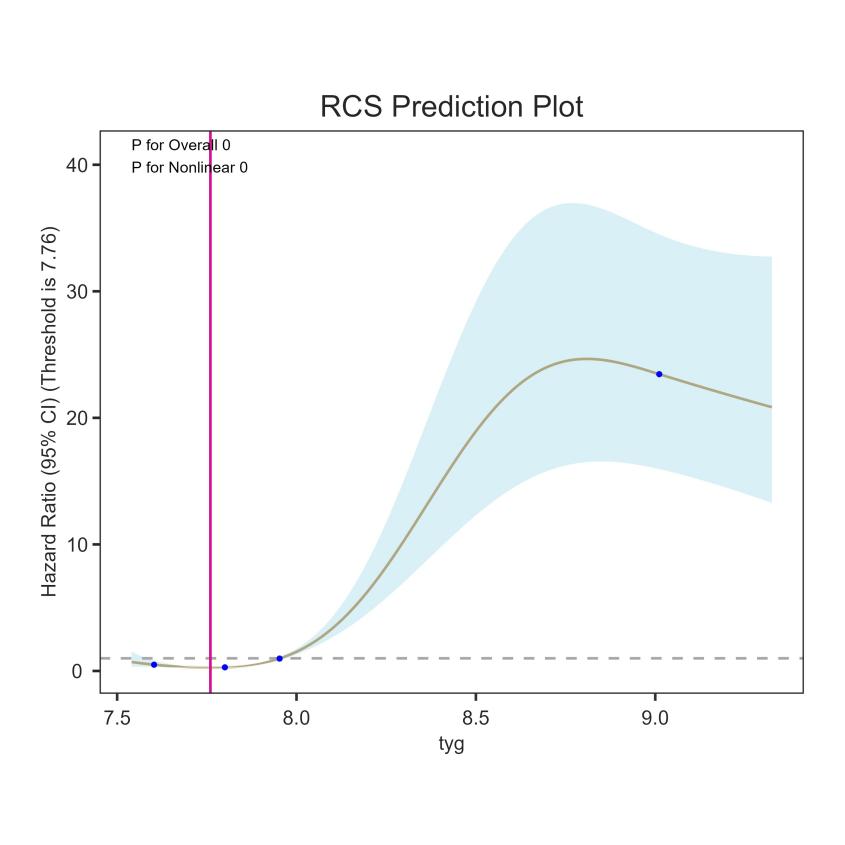
**

**Figure S5. RCS analysis of the TyG index and ICCE risk in the microsurgical treatment group**

**
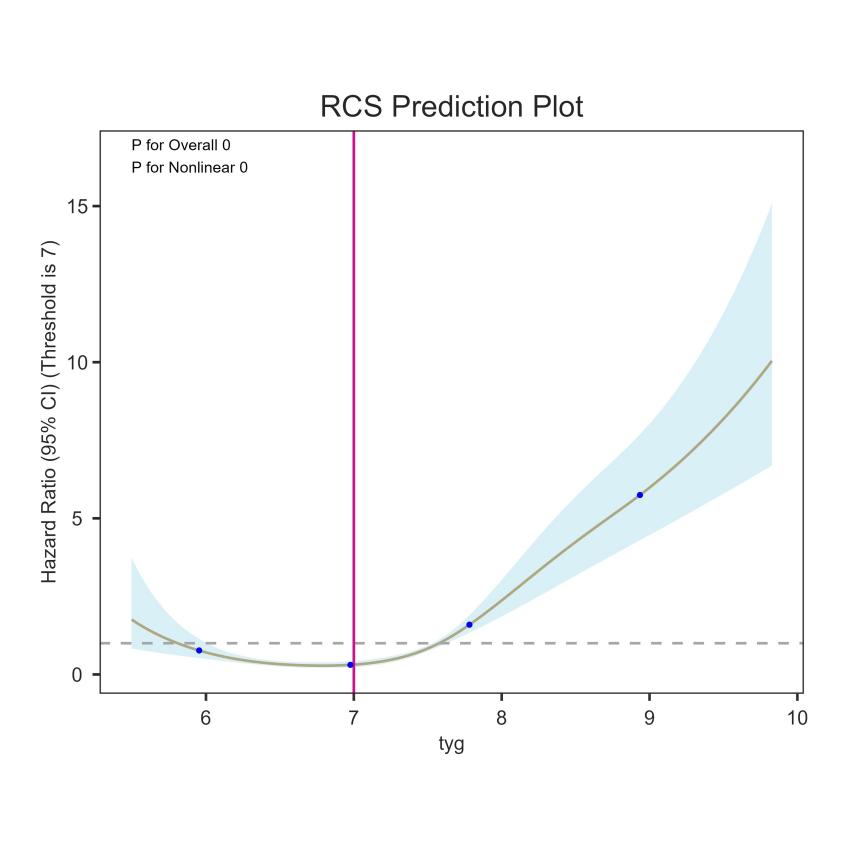
**

**Figure S6. RCS analysis of the TyG index at Beijing Tiantan Hospital**

**
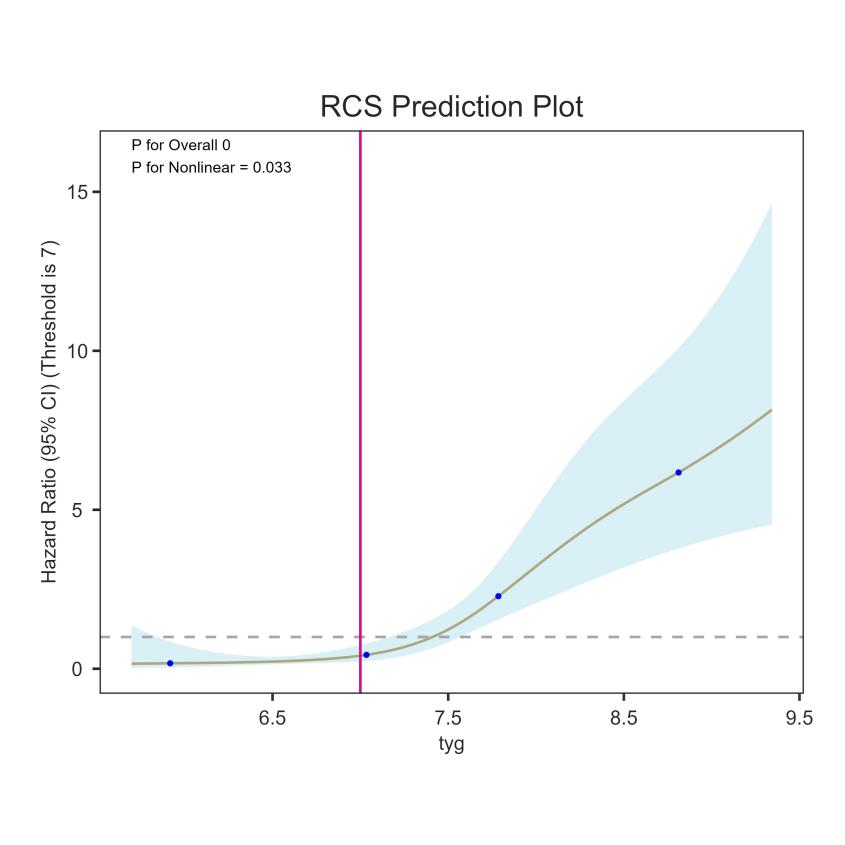
**

**Figure S7. RCS analysis of the TyG index at Fujian Provincial Hospital**

**
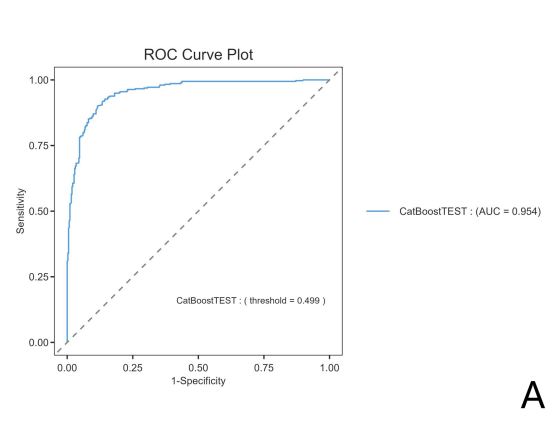

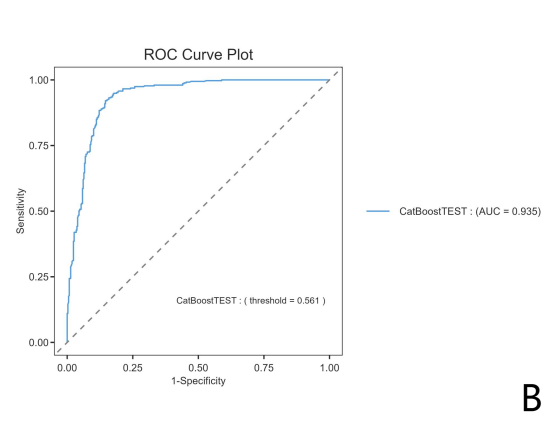
**

**
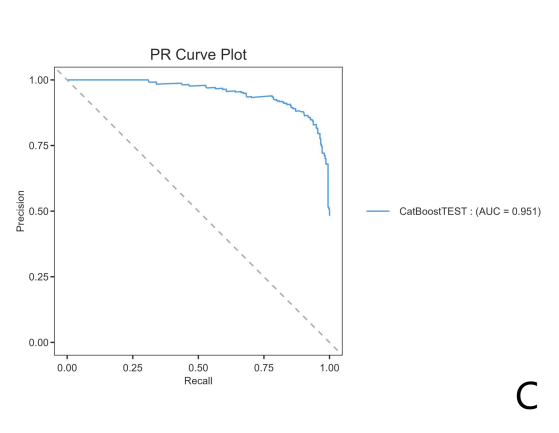

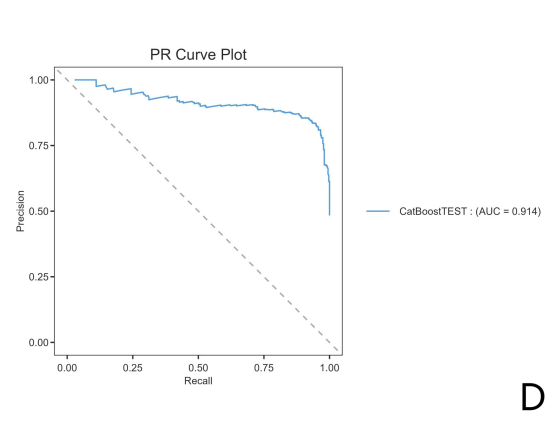
**

**
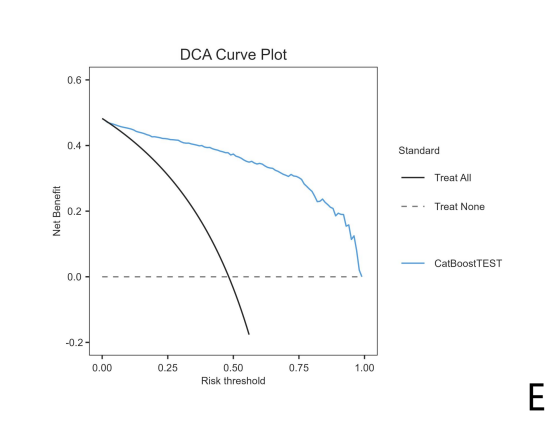

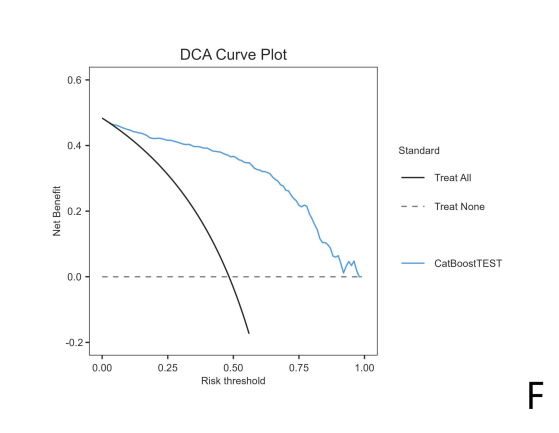

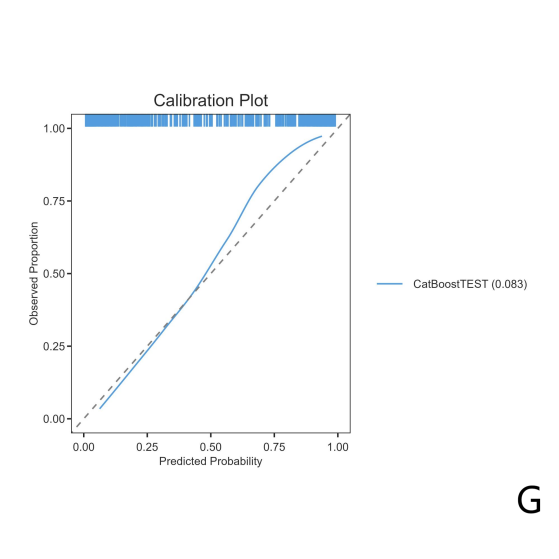

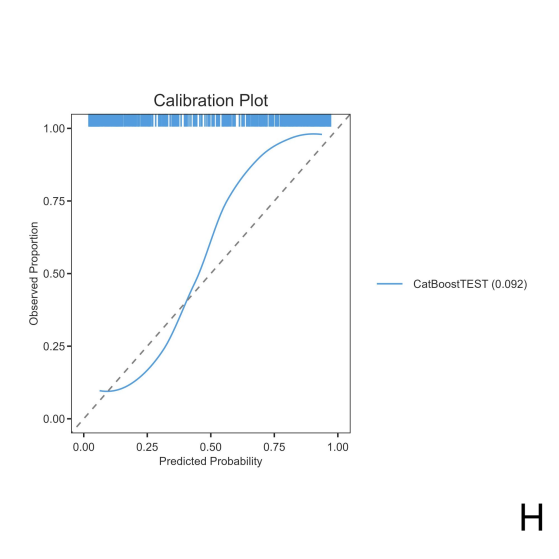
**

**Figure S8. ROC, PR, calibration, and DCA curves for ACS-specific and cerebral infarction-specific CatBoost models**

Performance evaluation of the CatBoost models for the two components of the composite endpoint.(A, C, E, G) Receiver operating characteristic (ROC), precision-recall (PR), decision curve analysis (DCA), and calibration curves for the cerebral infarction-specific model.(B, D, F, H) Receiver operating characteristic (ROC), precision-recall (PR), decision curve analysis (DCA), and calibration curves for the ACS-specific model.Both component-specific models demonstrated high discriminative ability (AUROC > 0.95), good calibration, and clear clinical utility across a wide range of risk thresholds.

**
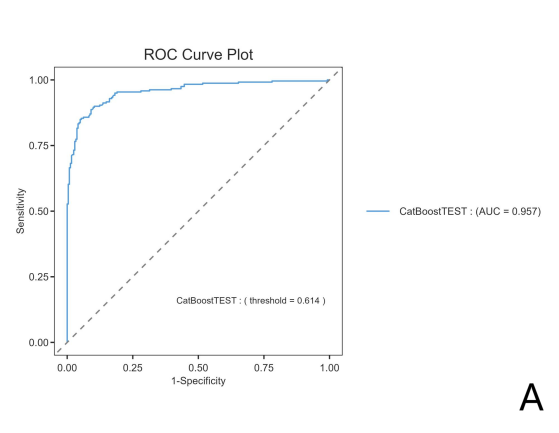

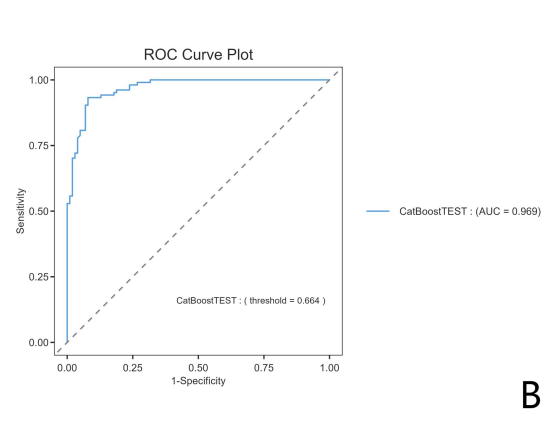
**

**
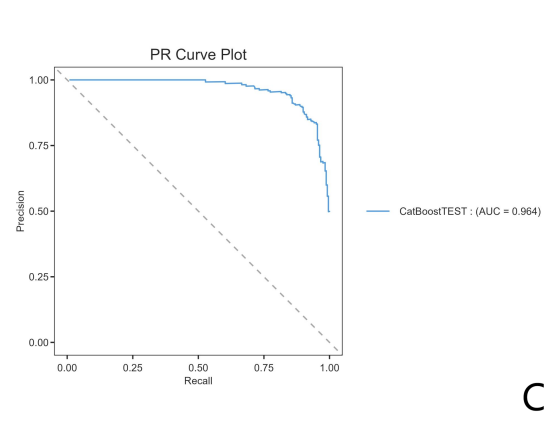

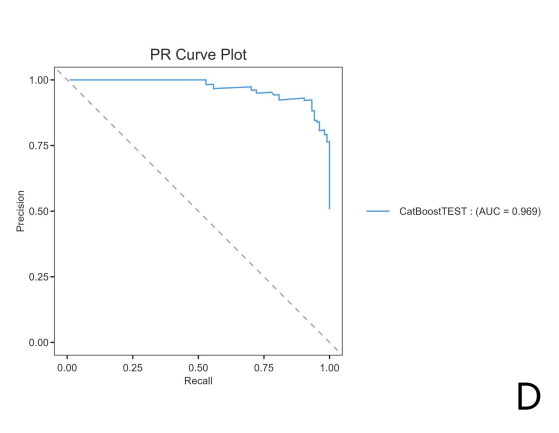
**

**
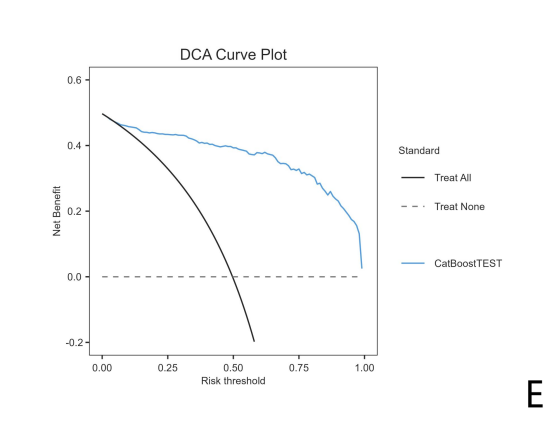

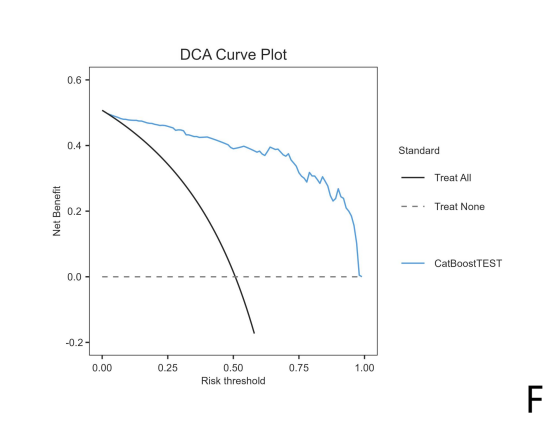
**

**
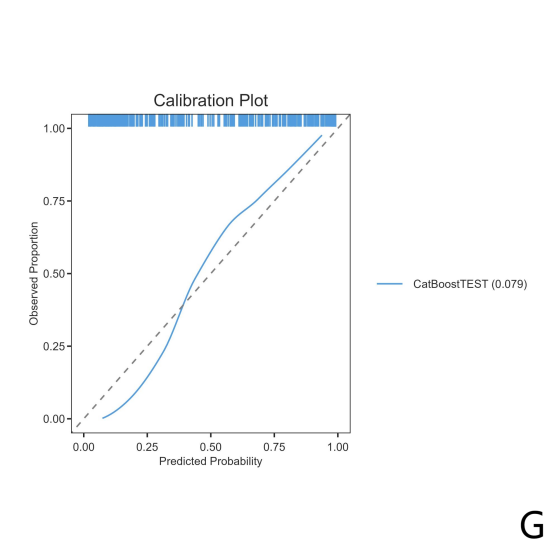

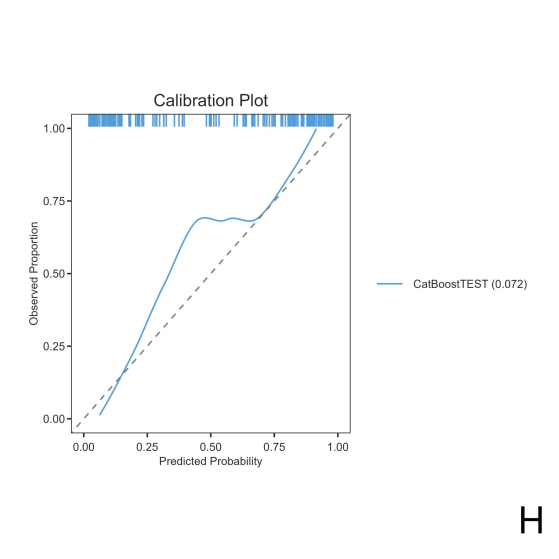
**

**Figure S9. ROC, PR, calibration, and DCA curves for center-specific CatBoost models**

Performance evaluation of the CatBoost models stratified by participating center.(A, C, E, G) Receiver operating characteristic (ROC), precision-recall (PR), decision curve analysis (DCA), and calibration curves for the model developed in the Beijing Tiantan Hospital cohort.(B, D, F, H) Receiver operating characteristic (ROC), precision-recall (PR), decision curve analysis (DCA), and calibration curves for the model developed in the Fujian Provincial Hospital cohort.Both center-specific models maintained high discriminative performance (AUROC > 0.95), good calibration, and clear clinical net benefit across a wide range of risk thresholds, supporting the robustness of the findings across different institutions.
